# *Staphylococcus epidermidis* prevents UV-induced skin aging by suppressing TLR3-mediated senescence

**DOI:** 10.3389/fimmu.2026.1796085

**Published:** 2026-05-12

**Authors:** Xinxin Wang, Mengke Wang, Jingya Yang, Wenbo Liu, Haidong Jia, Yuanyuan Chen, Yuping Lai

**Affiliations:** 1 Shanghai Frontiers Science Center of Genome Editing and Cell Therapy, School of Life Sciences, East China Normal University, Shanghai, China; 2 Shanghai Key Laboratory of Regulatory Biology, School of Life Sciences, East China Normal University, Shanghai, China; 3 Shanghai Jahwa United Co. Ltd, Shanghai, China; 4 Liwa institute of Skin Health, East China Normal University, Shanghai, China

**Keywords:** innate immunity, keratinocyte senescence, *S. epidermidis*, SASP factors, skin photoaging

## Abstract

Ultraviolet (UV) radiation is a major environmental driver of skin photoaging and induces keratinocyte senescence accompanied by the release of senescence-associated secretory phenotype (SASP) factors that promote dermal degeneration. However, whether and how skin commensal bacteria modulate UV-induced senescence remains incompletely understood. Here, we investigated the potential role of *Staphylococcus epidermidis*, a dominant epidermal commensal, in regulating UV-induced skin photoaging. In a murine photoaging model, intradermal or topical administration of a ≤10 kDa fraction derived from *S. epidermidis* culture supernatant attenuated epidermal hyperplasia, collagen degradation, and the expression of senescence-associated markers following long-term UV exposure. In keratinocytes, UVB irradiation induced reactive oxygen species accumulation, DNA damage, and robust production of SASP factors that promoted paracrine senescence in dermal fibroblasts. Treatment with *S. epidermidis* or its candidate bioactive lipopeptide component LP78 markedly reduced these responses. Genetic deletion or silencing of Toll-like receptor 3 (*Tlr3*) diminished UV-induced SASP factor production and fibroblast senescence, supporting a role of TLR3 in photoaging-associated inflammatory signaling. Mechanistically, *S. epidermidis* and LP78 activated TLR2 signaling to induce TNF receptor-associated factor 1 (TRAF1), a negative regulator of TLR3, thereby suppressing TLR3-mediated SASP production. Collectively, these findings identify a potential microbiota-innate immune regulatory axis in which *S. epidermidis*-derived factors restrain keratinocyte inflammatory senescence and attenuate UV-induced skin damage. This work highlights a potential role of commensal bacteria in limiting photoaging-associated inflammation.

## Introduction

Skin aging is broadly categorized into intrinsic aging, driven by genetically programmed and time-dependent processes, and extrinsic aging, which results from environmental stressors, most prominently ultraviolet (UV) radiation (1, 2). Photoaging, the predominant form of extrinsic aging, primarily affects chronically sun-exposed skin and is characterized by epidermal and dermal structural alterations, reduced elasticity, wrinkle formation, and pigmentary changes (3). Beyond cosmetic manifestations, photoaging compromises cutaneous barrier integrity and immune homeostasis, thereby increasing susceptibility to UV-associated disorders, including actinic keratosis and nonmelanoma skin cancer (4–6). Despite extensive investigation, the molecular mechanisms that coordinate UV-induced tissue damage with persistent inflammatory responses remain incompletely defined.

A hallmark of tissue aging is the accumulation of senescent cells, which acquires a senescence-associated secretory phenotype (SASP) (7–10). SASP factors include proinflammatory cytokines, chemokines, and matrix-remodeling enzymes that sustain local inflammation, promote extracellular matrix degradation, and propagate senescence to neighboring cells through paracrine signaling (7). In the skin, UV-induced oxidative stress activates signaling pathways such as NF-κB and MAPK, which drive SASP production and chronic inflammatory responses that accelerate photoaging progression (11–13). Increasing evidence further implicates that innate immune sensing pathways participate in these processes. For example, UV-induced accumulation of cytoplasmic DNA can activate the cGAS-STING pathway to promote senescence-associated inflammation, whereas inhibition of this pathway alleviates aging-related phenotypes in multiple tissues (14–16). In addition, RNA released from keratinocytes following UVB exposure has been shown to activate Toll-like receptor 3 (TLR3), leading to the production of proinflammatory cytokines such as IL-6 and TNFα (17). However, whether TLR3 directly regulates SASP factor production and paracrine senescence during skin photoaging remains incompletely understood.

The skin microbiome has emerged as an important regulator of cutaneous immune homeostasis. Among commensal microorganisms, *Staphylococcus epidermidis* (*S. epidermidis*) plays a prominent role in maintaining skin health by limiting pathogen colonization and modulating host immune responses (18–21). Factors derived from *S. epidermidis* have been shown to attenuate UV-induced inflammation, oxidative stress and tumorigenesis (17, 22). In our previous studies, we demonstrated that a ≤10 kDa fraction of *S. epidermidis* culture supernatant (≤10kDa *S. epi*) and its bioactive lipopeptide component LP78 suppress TLR3-mediated inflammatory signaling in keratinocytes during wound repair (23, 24). Whether this commensal-mediated regulation of innate immune signaling influences UV-induced cellular senescence and skin photoaging, however, remains unknown.

In this study, we investigate the role of *S. epidermidis* in regulating UV-induced keratinocyte senescence and SASP factor production. Our results suggest that UV exposure induces SASP factor expression in keratinocytes through a TLR3-mediated mechanism, which promotes paracrine senescence in dermal fibroblasts. In contrast, *S. epidermidis* and LP78 activate TLR2 signaling to induce TRAF1, a negative regulator of TLR3, thereby reducing TLR3-associated SASP factor production in keratinocytes. These findings support the existence of a commensal-driven regulatory mechanism that modulates innate immune signaling in keratinocytes and limits UV-induced inflammatory responses associated with skin photoaging.

## Results

### *Staphylococcus epidermidis* attenuates UV-induced skin photoaging *in vivo*

To examine whether *S. epidermidis* modulates UV-induced skin photoaging, bacterial culture supernatants were first fractionated by molecular weight and evaluated for cytotoxicity. Consistent with previous observations (25), the ≥10kDa fraction exhibited marked cytotoxic effects on keratinocytes and fibroblasts, whereas the ≤10kDa faction (≤10kDa *S. epi*) showed no detectable cytotoxicity (**Supplementary Figures 1A, B**). Therefore, the ≤10kDa *S. epi* fraction was used for subsequent analyses.

**Figure 1:**
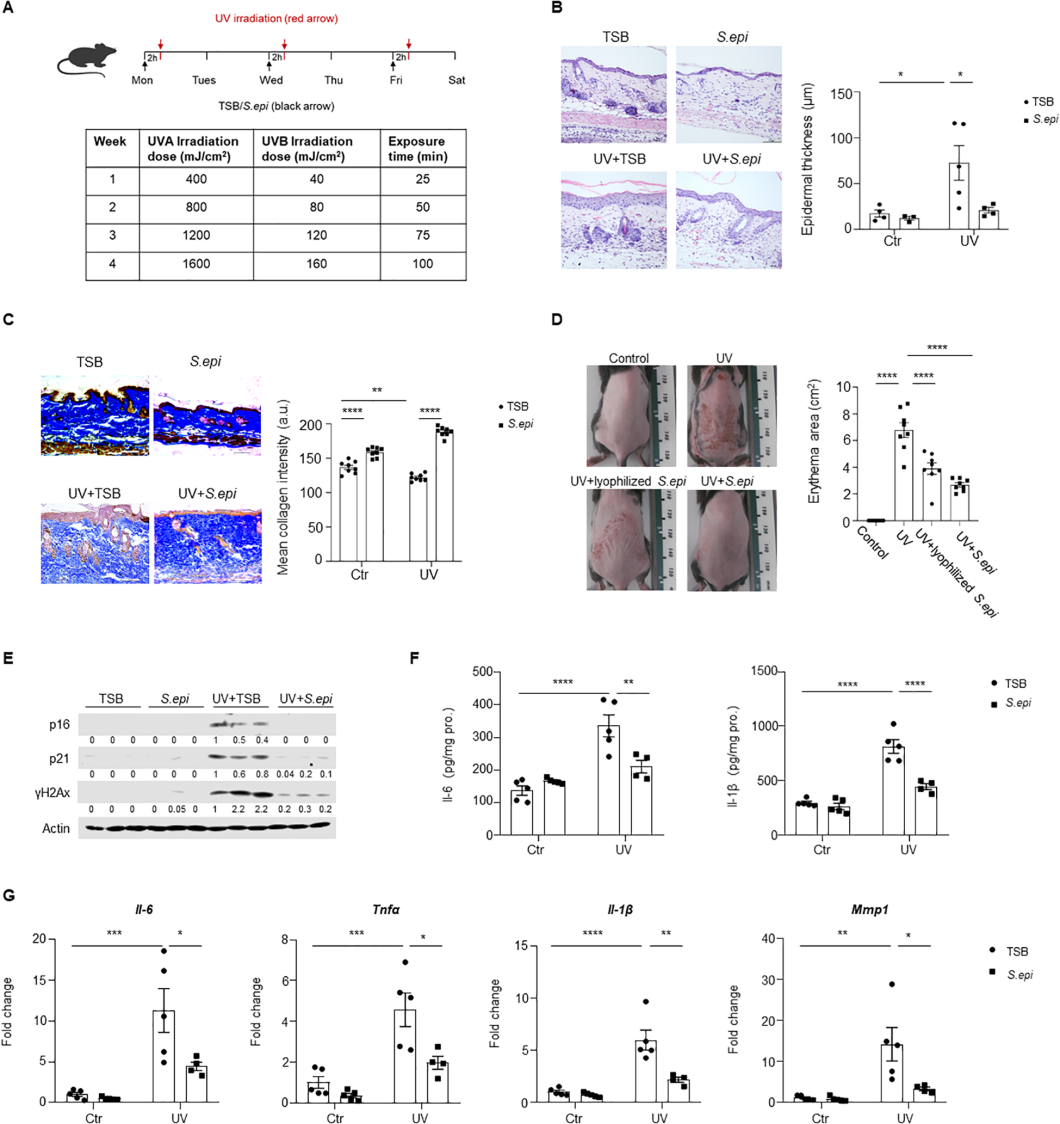
*Staphylococcus epidermidis* protects skin from photoaging in mice. **(A)** Schematic representation of the experimental design. A long-term photoaging model was established in wild-type C57BL/6 mice. Mice were divided into four groups: UV irradiation (red arrows) or no irradiation, with intradermal injections of either 100 μL Tryptic Soy Broth (TSB as a control) or 100 μg ≤10kDa *S. epi* (black arrows) 2 hours prior to each UV exposure **(B, C)**, or gels with or without ≤10kDa *S. epi* were applied topically **(D)**. UV irradiation was performed three times per week, with gradually increasing doses over four weeks. At the end of the experiment, skin tissues were collected for histological, western blotting, RT-PCR, and ELISA. **(B)** H&E-stained sections of dorsal skin from each experimental group. Epidermal thickness was quantified using ImageJ software. **(C)** Masson’s trichrome staining of dorsal skin, illustrating collagen content. Collagen intensity was quantified using ImageJ software. **(D)** Representative images of dorsal skin from mice topically treated with either lyophilized ≤10kDa *S. epi* or gel containing lyophilized ≤10kDa *S. epi*. Erythema area was quantified using ImageJ software. **(E)** Western blot analysis of P16, P21, and γH2Ax expression in dorsal skin samples. Densitometric analysis of protein bands were quantified by Image **(J, F)** ELISA quantification of Il-6 and Il-1β levels in dorsal skin tissues. **(H)** RT-PCR analysis of *Il-6*, *Tnfα*, *Il-1β*, and *Mmp1* expression, with β-actin as the internal control. Data represent mean ± SEM. Each point represents an individual mouse. All the experiments have been repeated twice. Statistical significances were determined by Two-way ANOVA. *p < 0.05, **p < 0.01, ***p < 0.001, ****p < 0.0001.

To assess the effects of ≤10kDa *S. epi in vivo*, a long-term UVA/UVB-induced photoaging model was established in mice (**Figure 1A**). Intradermal administration of ≤10kDa *S. epi* markedly reduced UV-induced epidermal hyperplasia and overall skin damage compared to vehicle-treated controls (**Figure 1B**, **Supplementary Figure 2A**). Masson’s trichrome staining demonstrated preservation of dermal collagen content in ≤10kDa *S. epi*-treated mice (**Figure 1C**). Comparable protective effects were observed following topical application of ≤10kDa *S. epi* formulated in a gel (**Figure 1D**). At the molecular level, UV-induced expression of senescence-associated markers p16, p21, and γH2Ax was markedly reduced in skin treated with ≤10kDa *S. epi* (**Figure 1E**, **Supplementary Figure 2B**). In parallel, the expression of SASP-associated factors, including Il-6, Tnfα, Il-1β, and Mmp1, was significantly decreased (**Figures 1F, G**). These data demonstrate that *S. epidermidis* attenuates UV-induced skin photoaging and associated senescence responses *in vivo*.

### *Staphylococcus epidermidis* selectively suppresses UVB-induced keratinocyte senescence

**Figure 2:**
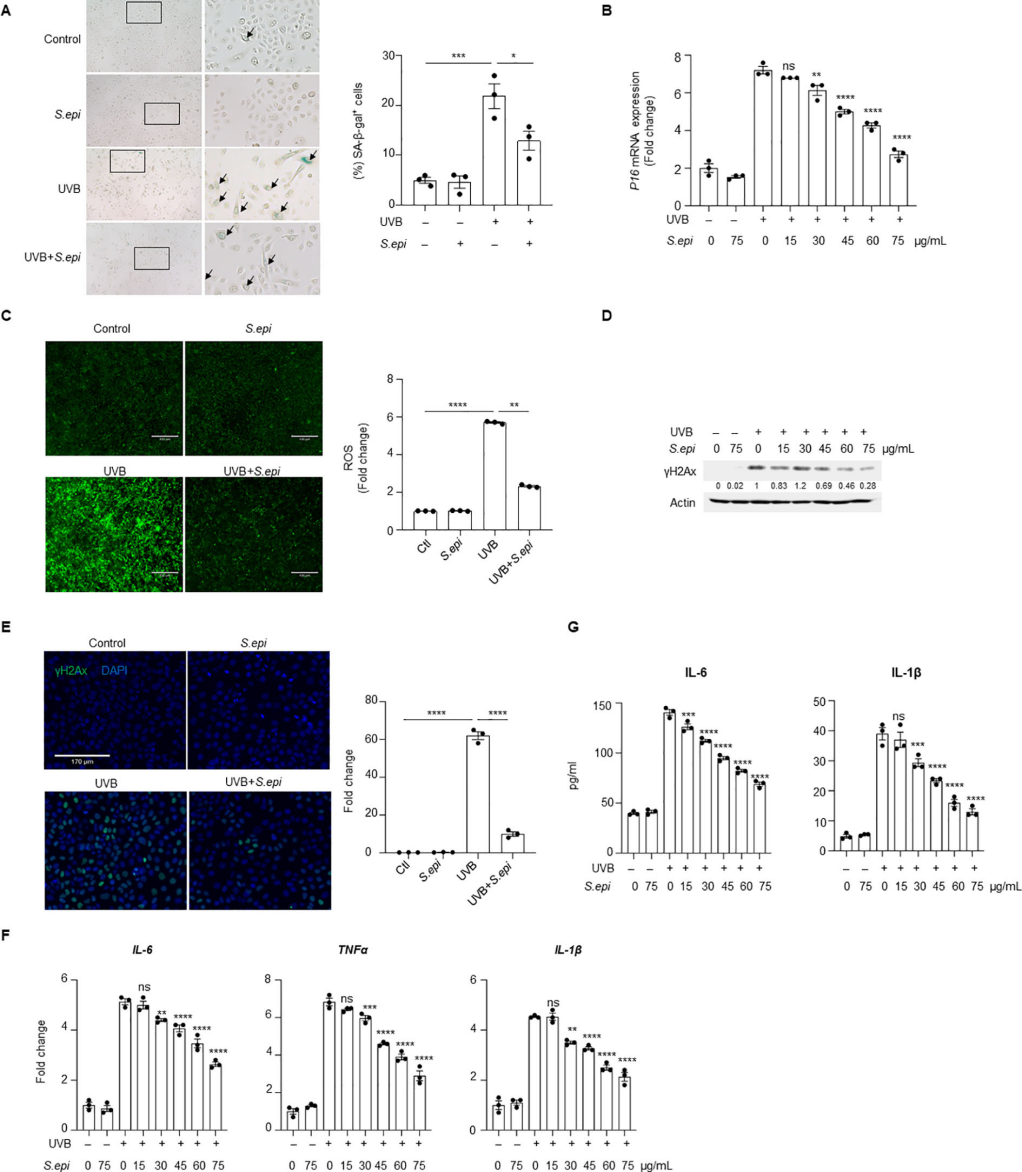
*Staphylococcus epidermidis* inhibits UVB-induced senescence in keratinocytes. **(A)** Neonatal human epidermal keratinocytes (NHEKs) at 90% confluence were irradiated with 10 mJ/cm^2^ UVB, followed by treatment with 75 μg/mL ≤10kDa *S. epi*. β-gal staining was performed 24 hours post-treatment. The proportion of senescent cells was quantified using Image **(J, B)** After UVB irradiation, different concentrations of ≤10kDa *S. epi* were added to NHEKs. RNA was isolated 24 hours later, and *P16* expression was measured by quantitative RT-PCR with β-actin as the internal control. **(C)** NHEKs at 90% confluence were irradiated with 10mJ/cm^2^ UVB, followed by treatment with 75μg/mL ≤10kDa *S. epi*. Six hours later, intracellular ROS were labeled with DCFH-DA and visualized using fluorescence microscope (excitation: 488 nm; emission: 525 nm), and fluorescence intensity was quantified using Image **(J, D)** NHEKs were treated with different concentrations of ≤10kDa *S. epi*. Six hours later, proteins were extracted for γH2Ax analysis, and densitometric analysis of protein bands were quantified by Image **(J)** Immunoblotting results are representative of three independent experiments performed with independent cultured keratinocytes. **(E)** NHEKs were irradiated with 10mJ/cm^2^ UVB and treated with 75μg/mL ≤10kDa *S. epi*. Six hours post-treatment, γH2Ax expression was visualized by immunofluorescence, and fluorescence intensity was quantified using Image **(J, F)** After UVB exposure (10mJ/cm^2^), NHEKs were treated with different concentrations of ≤10kDa *S. epi*. RNA was isolated 24 hours later, and the expressions of *IL-6*, *TNFα* and *IL-1β* was analyzed by quantitative RT-PCR using β-actin as internal control. **(G)** Following UVB irradiation (10mJ/cm^2^), NHEKs were treated with different concentrations of ≤10kDa *S. epi*. 24 hours later, cell culture supernatants were collected, and IL-6 and IL-1β secretion was measured by ELISA. Data represent mean ± SEM from independent biological replicates (*n* = 3). All the experiments have been repeated three times. Statistical significances were evaluated by One-way ANOVA. * *p* < 0.05, ** *p* < 0.01, *** *p* < 0.001, ****p < 0.0001, n.s., no significance.

Given the contributions of keratinocytes and fibroblasts to photoaging (10), we next examined whether ≤10kDa *S. epi* directly regulates senescence in these cell types. *In vitro* senescence models were established using primary keratinocytes and fibroblasts. Treatment with ≤10kDa *S. epi* did not significantly alter UVA-induced senescence in fibroblasts, as assessed by senescence-associated β-galactosidase (β-gal) staining and P16 expression (**Supplementary Figures 3A, B**). In contrast, ≤10kDa *S. epi* significantly reduced UVB-induced senescence in keratinocytes, with an approximately 40% decrease in β-gal positive cells (**Figure 2A**) and reduced P16 expression (**Figure 2B**). Treatment with ≤10kDa *S. epi* also reduced ROS accumulation by approximately 60% compared to UVB exposure alone (**Figure 2C**). Consistent with reduced oxidative stress, the ≤10kDa *S. epi* suppressed UVB-induced γH2Ax expression, as determined by immunoblotting and immunofluorescence analysis (**Figures 2D, E**, **Supplementary Figure 3C**). These results demonstrate that *S. epidermidis* selectively suppresses UVB-induced keratinocyte senescence.

Because senescent keratinocytes are a major source of SASP factors (7, 26), we next assessed whether ≤10kDa *S. epi* would regulate SASP factor expression. Quantitative RT-PCR analysis showed that ≤10kDa *S. epi* significantly reduced UVB-induced mRNA expression of the selected SASP-associated cytokines *IL-6*, *TNFα*, and *IL-1β* in keratinocytes (**Figure 2F**). Consistently, ELISA measurements demonstrated dose-dependent reduction in IL-6 and IL-1β secretion, with maximal inhibition of approximately 40% and 78%, respectively, at 50 μg/mL (**Figure 2G**). These data indicate that *S. epidermidis* suppresses both transcriptional induction and secretion of SASP factors in UVB-induced senescent keratinocytes.

### *Staphylococcus epidermidis* indirectly limits fibroblast senescence by suppressing keratinocyte-derived SASP factors

**Figure 3:**
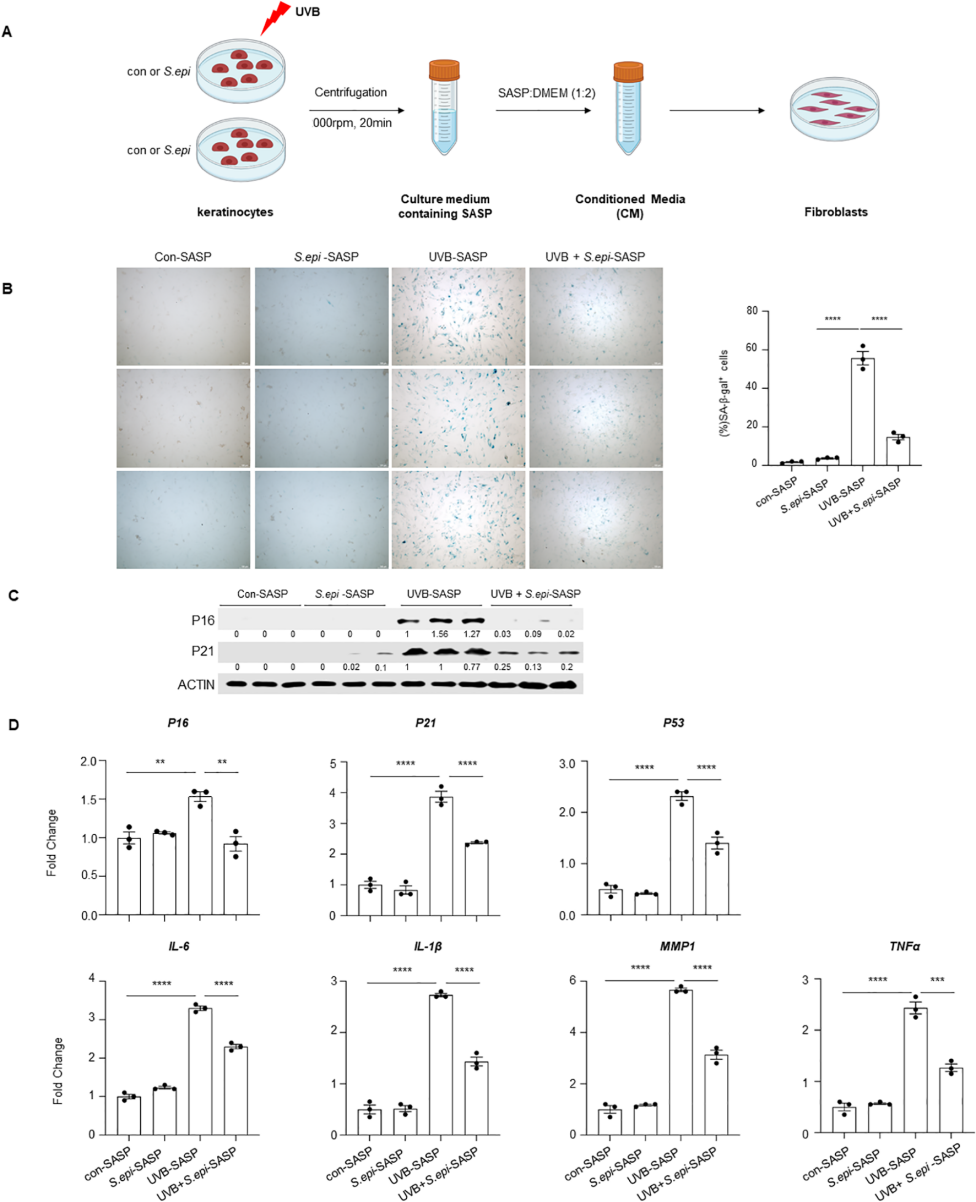
*Staphylococcus epidermidis* inhibits fibroblast senescence by inhibiting SASP secretion from UVB-irradiated keratinocytes. **(A)** Schematic representation of the experimental design. NHEKs were cultured to 90% confluence and then exposed to 10 mJ/cm^2^ UVB for induction. Subsequently, 75μg/mL of ≤10kDa *S. epi* was added to coculture for 48 hours. The conditioned medium containing SASP factors was collected by centrifugation at 2, 000 rpm for 20 minutes, mixed with fresh DMEM at a 1:2 ratio, and the final serum concentration was adjusted to 10%. The mixed medium was used to culture primary human fibroblasts for 48 hours. **(B)** Fibroblasts were stained with senescence-associated β-gal, and the percentage of senescent cells was quantified by Image **(J, C)** Protein levels of P16 and P21 in fibroblasts were analyzed by western blotting. Densitometric analysis of protein bands were quantified by Image **(J, D)** RT-PCR analysis of RNA isolated from fibroblasts was performed to assess the expression of *P16, P21*, *P53*, *TNFα*, *IL-6*, *IL-1β*, and *MMP1*, with β-actin as the internal control. Con-SASP, SASP collected from control NHEKs; *S.epi*-SASP, SASP collected from NHEKs treated with 75μg/mL of ≤10kDa *S.epi*; UVB-SASP, SASP collected from NHEKs treated with UVB; UVB+*S.epi*-SASP, SASP collected from NHEKs treated with UVB and 75μg/mL of ≤10kDa *S.epi*. Data represent mean ± SEM with *n* = 3. All the experiments have been repeated three times. Statistical significances were analyzed by One-way ANOVA. **p < 0.01, *** *p* < 0.001, ****p < 0.0001.

To determine whether keratinocyte-derived SASP factors could promote fibroblast senescence, conditioned media collected from UVB-exposed keratinocytes were applied to fibroblast cultures (**Figure 3A**). Conditioned media induced fibroblast senescence in a dilution-dependent manner, with maximal effects observed at a 1:2 ratio of conditioned medium to fresh medium (**Supplementary Figures 4A, B**). Using this ratio, conditioned media from UVB-exposed keratinocytes treated with ≤10kDa *S. epi* induced significantly less fibroblast senescence than media from untreated keratinocytes, as evidenced by an approximately 65% reduction in senescence-associated β-gal-positive fibroblasts (**Figure 3B**). Consistently, expression of the senescence markers P16 and P21 was reduced in fibroblasts exposed to conditioned media from ≤10kDa *S. epi*-treated keratinocytes (**Figure 3C**, **Supplementary Figure 4C**). Quantitative RT-PCR analysis further revealed decreased expression of senescence-associated genes and selected SASP factors in these fibroblasts (**Figure 3D**). These results demonstrate *S. epidermidis* indirectly limits fibroblast senescence by suppressing SASP factor production in UVB-exposed keratinocytes.

### LP78 is a candidate bioactive *Staphylococcus epidermidis* component that suppresses skin senescence

**Figure 4:**
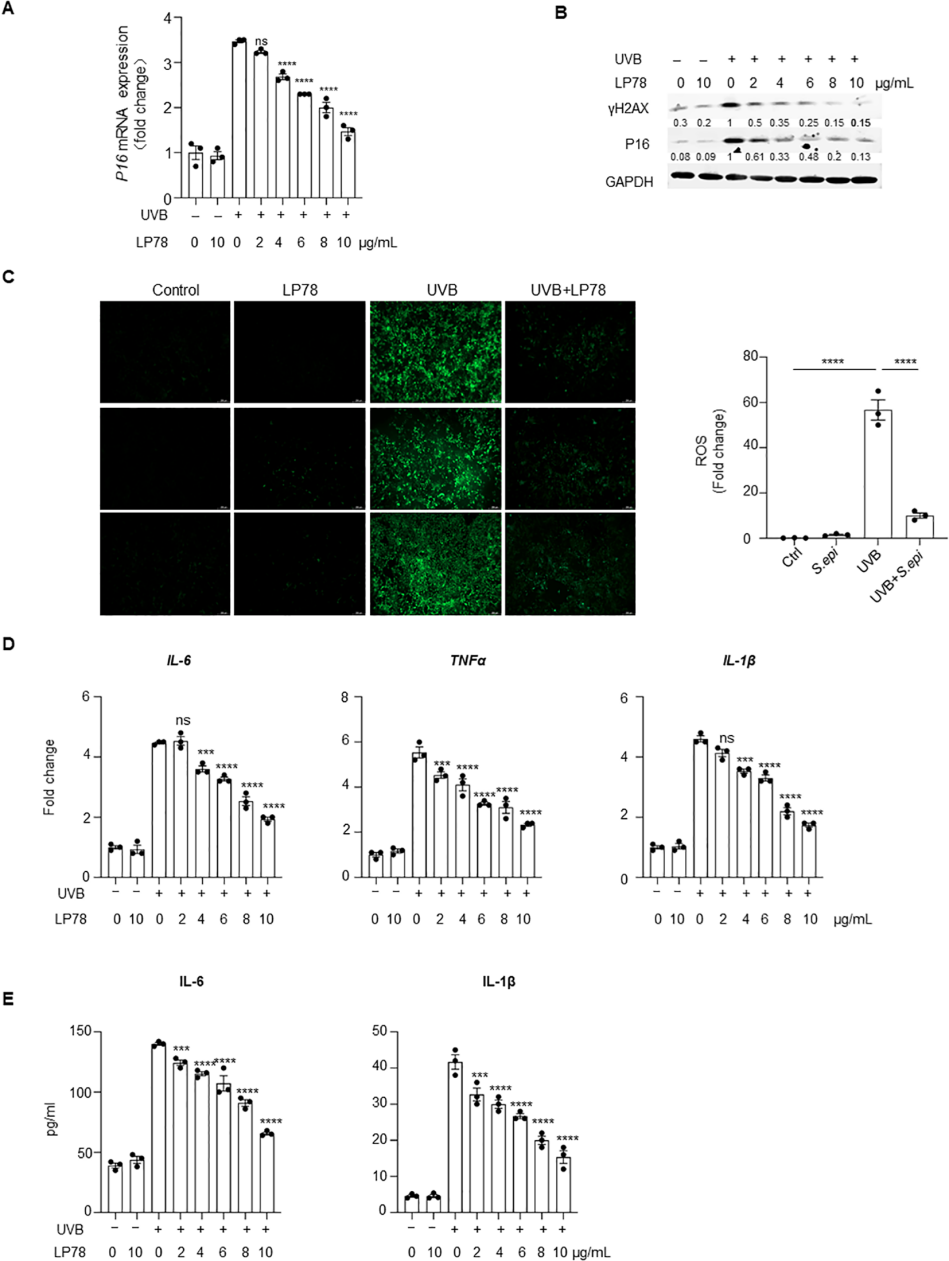
LP78 inhibits UVB-induced keratinocyte senescence. **(A)** NHEK at 90% confluency were irradiated with 10mJ/cm^2^ UVB, followed by treatment with different concentrations of LP78. RNA was extracted 24 hours later, and *P16* expression was detected by RT-PCR, with β-actin as the internal control. **(B)** NHEKs at 90% confluency were irradiated with 10mJ/cm^2^ UVB and treated with different concentrations of LP78. Cell lysates were collected after 24 hours, and P16 and γH2Ax levels were analyzed by western blotting. Densitometric analysis of protein bands were quantified by Image **(J, C)** NHEKs at 90% confluency were irradiated with 10mJ/cm^2^ UVB and treated with 10μg/mL LP78. Six hours post-treatment, intracellular ROS were visualized using fluorescence microscope and the intensity was quantified using Image **(J, D)** NHEK at 90% confluency were irradiated with 10mJ/cm^2^ UVB and treated with different concentrations of LP78. RNA was extracted 24 hours later, and the expression of *IL-6*, *TNFα* and *IL-1β* was measured by RT-PCR, with β-actin as the internal control. **(E)** NHEKs at 90% confluency were irradiated with 10mJ/cm^2^ UVB and treated with different concentrations of LP78. After 24 hours, culture supernatants were collected, and the secretion of IL-6 and IL-1β was quantified by ELISA. Data represent mean ± SEM with *n* = 3. All the experiments have been repeated three times. Statistical significances were analyzed by One-way ANOVA. *** *p* < 0.001, ****p < 0.0001, n.s., no significance.

We next examined whether lipopeptide 78 (LP78), a previously identified bioactive component of ≤10kDa *S. epi* (23), could exhibit anti-senescent activity. LP78 showed no detectable cytotoxicity in keratinocytes at concentrations up to 10 μg/mL (**Supplementary Figure 5A**). Treatment with LP78 significantly reduced UV-induced P16 expression (**Figures 4A, B**, **Supplementary Figure 5B**), γH2AX accumulation (**Figure 4B**, **Supplementary Figure 5B**), and intracellular ROS production (**Figure 4C**), comparable to the effects observed with ≤10kDa *S. epi*. In addition, LP78 suppressed both the expression and secretion of IL-6, TNFα, and IL-1β (**Figures 4D, E**). These data suggest that LP78 acts as a bioactive mediator of *S. epidermidis*-dependent suppression of keratinocyte senescence.

### *Staphylococcus epidermidis* suppresses UV-induced photoaging through TLR3-mediated signaling

**Figure 5:**
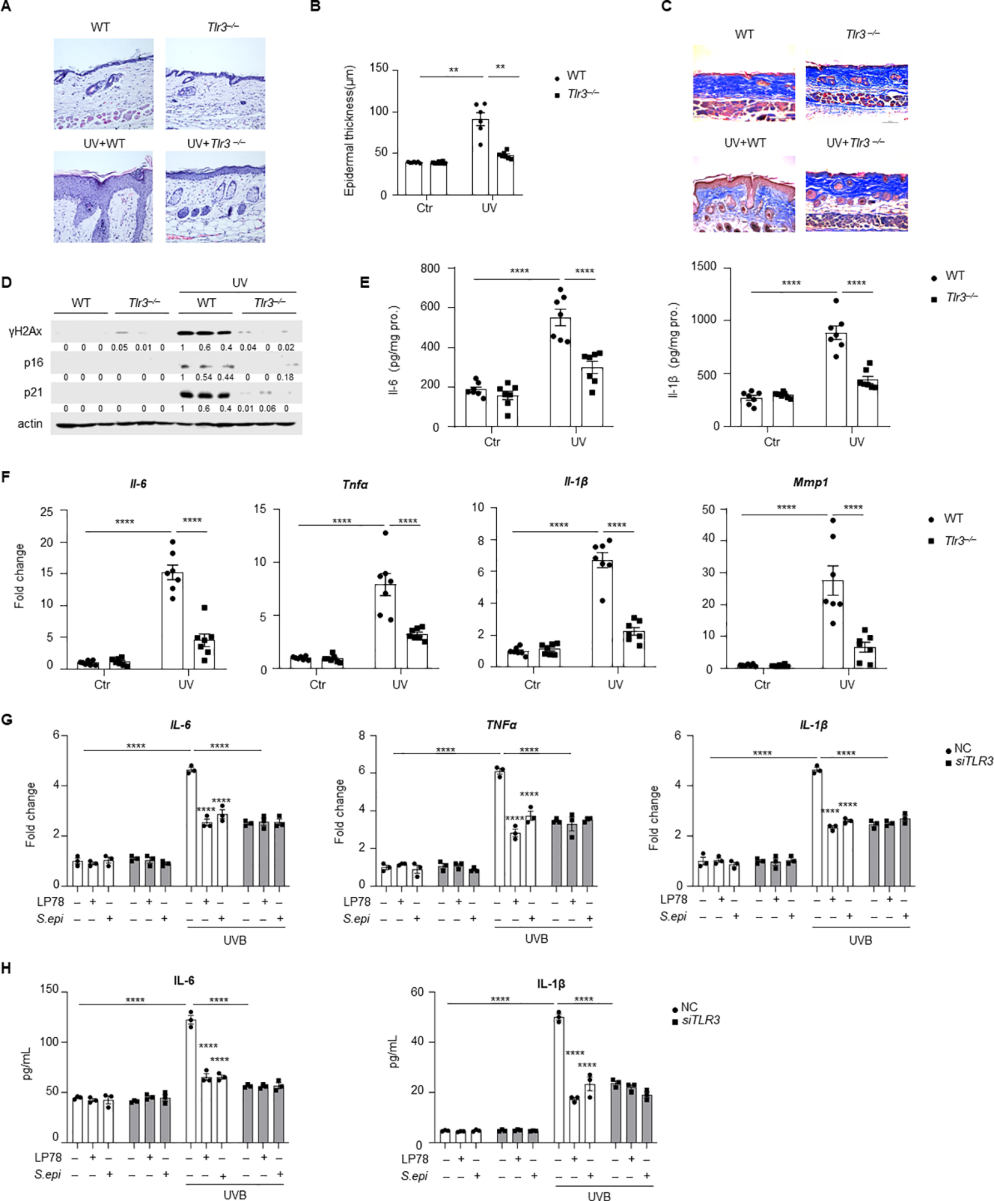
UV-induced skin photoaging is dependent on TLR3 activation, and *Staphylococcus epidermidis* and LP78 inhibit TLR3-mediated SASP factors. **(A)** A long-term photoaging model was established using WT and *Tlr3^−/−^* C57BL/6 mice (*n* = 5-7). The dorsal skin of each mouse was divided into upper (shield with tin foil) and lower (exposed) regions. The lower region was irradiated with UV as shown in **Figure 1A**. Representative photographs of dorsal skin were taken at the end of the experiment for HE staining. **(B)** Epidermal thickness of dorsal skin from mice treated as **(A)** was quantified using Image J software. **(C)** Masson’s trichrome staining of dorsal skin from mice treated as **(A)** was performed to evaluate dermal collagen content. **(D)** Western blot analysis of P16, P21 and γH2Ax protein expression in dorsal skin tissues from mice treated as **(A)**. Densitometric analysis of protein bands were quantified by Image **(J, E)** ELISA measurement of Il-6 and Il-1β in dorsal skin tissues from mice treated as **(A)**. **(F)** RT-PCR analysis of *Il-6*, *Tnfα*, *Il-1β* and *Mmp1*, with β-actin as an internal control. **(G)** NHEKs with or without *TLR3* silencing were irradiated with 10mJ/cm^2^ UVB at 90% confluence. Following UVB exposure, cells were treated with 75μg/mL of ≤10kDa *S. epi* or 10μg/mL of LP78. RNA was extracted 12h hours post-treatment, and *IL-6*, *TNFα* and *IL-1β* mRNA expression was assessed by RT-PCR, with β-Actin as a reference gene. **(I)** Supernatants were collected from the same cultures in **(G)**, and the secretion of IL-6 and IL-1β was measured by ELISA. Data represent mean ± SEM with *n* = 3-7. All the experiments have been repeated twice or three times. Statistical significances were analyzed by Two-way ANOVA. **p < 0.01, ****p < 0.0001.

Activation of pattern recognition receptor is a key driver of SASP production during UV-induced skin aging (7). To assess the role of TLR3 in UV-induced photoaging, long-term UV exposure was performed in wild-type (WT) and *Tlr3^−/−^* mice. UV irradiation induced epidermal thickening, inflammatory cell infiltration, collagen degradation, and increased expression of senescence markers in WT mice. These changes were markedly attenuated in *Tlr3^−/−^* mice (**Figures 5A–D**, **Supplementary Figures 6A, B**). Quantitative RT-PCR and ELISA analyses revealed significantly reduced expression and production of SASP-associated factors, including Il-6, Tnfα, Il-1β, and Mmp1, in UV-exposed *Tlr3^−/−^* skin compared to WT controls (**Figures 5E, F**). These data demonstrate that TLR3 is a key mediator linking UV exposure to skin photoaging.

To determine whether keratinocyte TLR3 would regulate paracrine senescence, keratinocytes were transfected with TLR3-targeting siRNA (siTLR3) prior to UVB exposure (**Supplementary Figure 6C**). TLR3 silencing significantly reduced UVB-induced senescence in keratinocytes (**Supplementary Figure 6D**), and conditional media from UVB-exposed siTLR3-transfected keratinocytes induced significantly less senescence in dermal fibroblasts than media from control keratinocytes, as evidenced by an approximately 40% reduction in β-gal-positive fibroblasts (**Supplementary Figures 6E, F**). Consistent with this, fibroblasts cultured with conditioned media from siTLR3-transfected keratinocytes exhibited reduced expression of P16, P21, and P53 at both mRNA and protein levels (**Supplementary Figures 6G–I**).

To determine whether *S. epidermidis* could regulate TLR3-mediated senescence signaling, primary human keratinocytes with or without *TLR3* silencing were subjected to UVB irradiation and subsequently treated with the ≤10kDa *S. epi* fraction or LP78. In WT keratinocytes, both treatments significantly reduced UVB-induced expression and secretion of IL-6, TNFα, and IL-1β. In contrast, neither ≤10kDa *S. epi* nor LP78 further decreased the levels of these SASP factors in *TLR3*-silenced keratinocytes (**Figures 5G, H**). These results demonstrate that *S. epidermidis* and LP78 suppress TLR3-driven SASP factor production in keratinocytes.

### *Staphylococcus epidermidis* and LP78 suppresses SASP factor production via a TLR2-TRAF1 pathway

**Figure 6:**
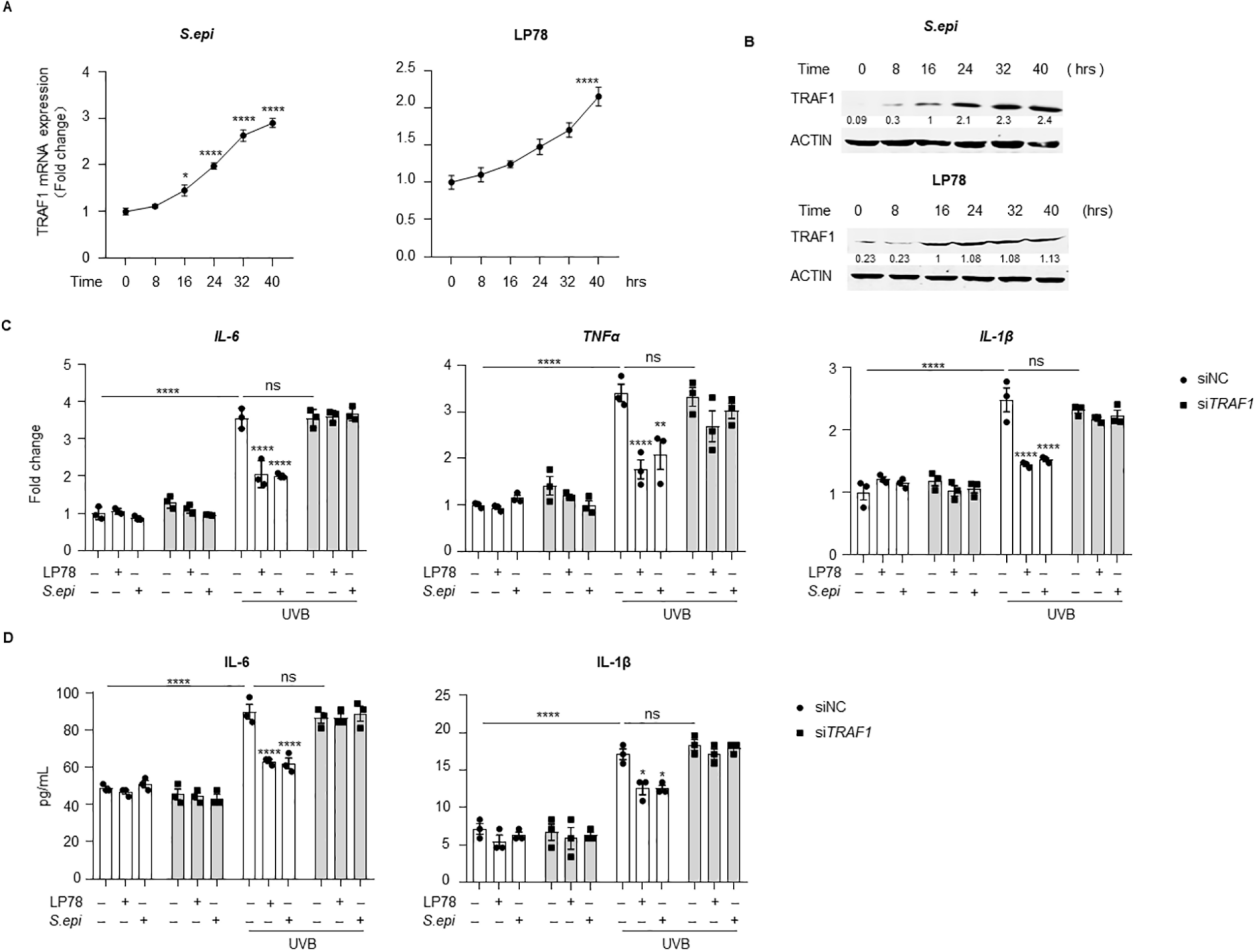
*Staphylococcus epidermidis* and LP78 inhibit SASP factor expression through TRAF1 induction. **(A)** NHEKs were treated with 75μg/mL of ≤10kDa *S. epi* or 10μg/mL of LP78. RNA was extracted at different time points, and *TRAF1* expression was assessed by RT-PCR, with β-actin as the internal control. **(B)** Western blot analysis of TRAF1 in NHEKs treated as in **(A)**. Densitometric analysis of protein bands were quantified by Image **(J, C)** NHEKs were transfected with *siTRAF1* using jetPRIME at 60% confluency. After 24 hours, the cells were exposed to 10mJ/cm^2^ UVB and treated with 75μg/mL of ≤10kDa *S. epi* or 10μg/mL of LP78. RNA was extracted after 12 hours post-treatment, and the expression of *IL-6*, *TNFα* and *IL-1β* was analyzed by RT-PCR, with β-actin as the internal reference. **(C)**The supernatant from the same culture in **(B)** was analyzed by ELISA to measure IL-6 and IL-1β secretion. Data represent mean ± SEM with *n* = 3. All the experiments have been repeated three times. Statistical significances were analyzed by Two-way ANOVA. *p < 0.05, ****p < 0.0001, n.s., no significance.

Next, we investigated the molecular mechanism by which *S. epidermidis* suppresses TLR3-mediated SASP factor production in keratinocytes. TRAF1 is a known negative regulator of TLR3 signaling (24). To determine whether TRAF1 mediates the inhibitory effects of *S. epidermidis* and LP78 on SASP factor production, we first examined TRAF1 expression in keratinocytes treated with ≤10kDa *S. epi* or LP78. Both treatments induced TRAF1 expression in a time-dependent manner over a 40-hour period (**Figures 6A, B**, **Supplementary Figure 7A**). Consistent with this induction, knockdown of *TRAF1* completely abolished the inhibitory effects of the ≤10kDa *S. epi* fraction or LP78 on UVB-induced expression and secretion of IL-6, TNFα, and IL-1β in keratinocytes (**Figures 6C, D**, **Supplementary Figures 7B, C**), demonstrating that TRAF1 is required for this suppression.

**Figure 7:**
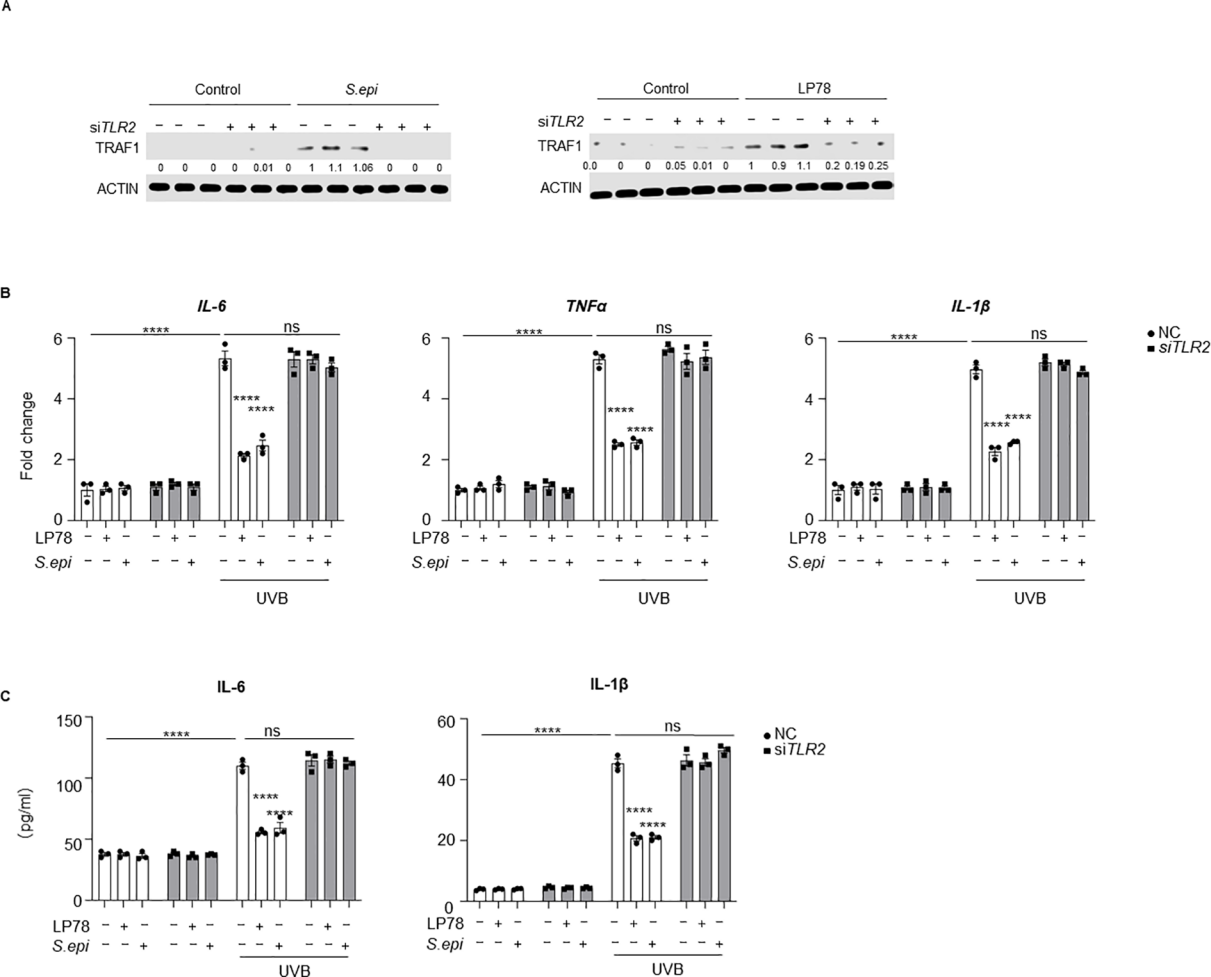
*Staphylococcus epidermidis* and LP78 induce TRAF1 dependent on TLR2. **(A)** NHEKs were transfected with *siTLR2* using jetPRIME at 60% confluency. After 24 hours, the cells were treated with 75μg/mL of ≤10kDa *S. epi* or 10μg/mL of LP78. Cell lysates was extracted after 12 hours post-treatment, and the expression of TRAF1 was analyzed Western blotting. Densitometric analysis of protein bands were quantified by Image **(J, B)** NHEKs were transfected with *siTLR2* using jetPRIME at 60% confluency. After 24 hours, the cells were exposed to 10mJ/cm^2^ UVB and treated with 75μg/mL of ≤10kDa *S. epi* or 10μg/mL of LP78. RNA was extracted after 12 hours post-treatment, and the expression of *IL-6*, *TNFα* and *IL-1β* was analyzed by RT-PCR, with β-actin as the internal reference. **(C)**The supernatant from the same culture in **(B)** was analyzed by ELISA to measure IL-6 and IL-1β secretion. Data represent mean ± SEM with *n* = 3. All the experiments have been repeated three times. Statistical significances were analyzed by Two-way ANOVA. ****p < 0.0001, n.s., no significance.

Because induction of TRAF1 expression by ≤10kDa *S. epi* is TLR2-dependent (24), we next assessed the role of TLR2 in this regulatory pathway. TLR2 silencing markedly reduced the induction of TRAF1 following ≤10kDa *S. epi* or LP78 stimulation (**Figure 7A**, **Supplementary Figures 7D, E**), even though control siRNA transfection modestly increased basal TRAF1 expression that reflects transfection-associated cellular stress. Accordingly, *TLR2* knockdown prevented both treatments from suppressing UVB-induced IL-6, TNFα, and IL-1β expression and secretion in keratinocytes (**Figures 7B, C**). Together, these data suggest that *S. epidermidis* and LP78 suppress UV-induced SASP factor production in keratinocytes and that this effect is associated with TLR2-TRAF1 signaling.

## Discussion

The role of skin commensal microbiota in regulating cutaneous aging remains incompletely understood. In this study, we provide evidence that *S. epidermidis*, a dominant epidermal commensal, functions as a regulator of UV-induced skin photoaging. Our findings indicate that low-molecular weight components derived from *S. epidermidis*, particularly the candidate bioactive lipopeptide LP78, attenuate keratinocyte senescence, suppress the production of selected SASP factors, thereby limiting paracrine senescence in dermal fibroblasts. Mechanistically, these effects appear to involve TLR2-mediated induction of TRAF1, which acts as a negative regulator of TLR3-mediatged inflammatory signaling. Together, these findings support the existence of a microbiota-innate immune regulatory axis that modulates keratinocyte inflammatory responses and may limit inflammatory senescence associated with UV-induced skin damage.

A key observation of this study is that *S. epidermidis* preferentially modulates UVB-induced keratinocyte senescence rather than directly acting on fibroblasts. Keratinocytes are the primary UV-responsive cells in the epidermis and are a major source of SASP factors following genotoxic stress (7). Consistent with previous reports (27–30), UVB exposure induced robust expression and secretion of pro-inflammatory cytokines, including IL-6, TNF-α, and IL-1β, in keratinocytes. Treatment with *S. epidermidis* or LP78 markedly suppressed the expression of these factors. Although fibroblasts were relatively insensitive to direct modulation by *S. epidermidis*, they exhibited pronounced senescence when exposed to conditioned media from UVB-treated keratinocytes. Importantly, suppression of SASP production in keratinocytes substantially attenuate fibroblast senescence in this paracrine system. These findings support a model in which epidermal inflammatory senescence contributes to downstream dermal aging processes.

Our data also implicate TLR3 as a critical regulator of UV-induced SASP expression and photoaging-associated tissue alterations. Genetic ablation or silencing of *Tlr3* markedly reduced epidermal hyperplasia, inflammatory infiltration, collagen degradation, and expression of senescence markers in UV-exposed skin. At the cellular level, TLR3 activation promoted senescent gene expression and SASP factor production in keratinocytes and enhanced the ability of keratinocyte-derived factors to induce fibroblast senescence. These observations are consistent with emerging evidence that nucleic acid-sensing pathways, including TLR3 and cGAS-STING axis, involve in senescence-associated inflammation following cellular stress (8, 16, 31, 32). Our findings therefore support a role of TLR3 not only in innate immune sensing but also in the regulation of inflammatory pathways associated with tissue aging.

An additional mechanistic insight from this study is the identification of a commensal-associated pathway that counteracts TLR3-mediated inflammatory signaling. We found that *S. epidermidis* and LP78 induced TRAF1 expression in a TLR2-depdent manner and that TRAF1 was required for the suppression of UV-induced SASP factor expression. TRAF1 has previously been described as a negative regulator of TLR3 signaling (24), and our results suggest that this inhibitory function may extend to the regulation of senescence-associated inflammatory responses during photoaging. This proposed TLR2-TRAF1-TLR3 regulatory axis provides a conceptual framework through which beneficial skin commensals may buffer excessive damage-induced inflammation while maintaining innate immune competence.

In addition to suppressing inflammatory pathways, *S. epidermidis* and LP78 significantly reduced ROS accumulation and DNA damage in UVB-exposed keratinocytes. Although the underlying mechanisms were not directly investigated here, these findings raised the possibility that commensal-derived signals may influence cellular redox homeostasis during photoaging. Oxidant stress plays a central role in UV-induced skin aging, and antioxidant pathways such as the Nrf2-dependent stress response are known to limit oxidative stress (12, 33–35). Future studies will be required to determine whether *S. epidermidis* or LP78 engage these protective pathways and how redox regulation intersects with SASP signaling during senescence progression.

Our analyses focused on several representative SASP components, including IL-6, TNF-α, and IL-1β; however, UV-induced SASPs encompass a much broader network of inflammatory cytokines, chemokines, and matrix-remodeling enzymes (28–30). Comprehensive transcriptomic and secretome profiling will therefore be necessary to define the full spectrum of SASP factors influenced by *S. epidermidis*-derived signals. In addition, although keratinocyte-derived SASP factors clearly drive fibroblast senescence in our experimental system, the relative contribution of individual cytokines remains to be determined and will require targeted genetic or biochemical approaches.

Moreover, several additional questions remain to be addressed. TLR3 expression has been reported to increase in senescent cells and aged tissues (36–38), and we observed elevated TLR3 levels in senescent fibroblasts and aged mouse skin (Data not shown). Whether TLR3 is involved in chronological aging independently of UV exposure remains unclear and will require investigation using aging-specific models and cell-type-restricted genetic approaches. Furthermore, although LP78 recapitulates several protective effects of *S. epidermidis*, it is unlikely to represent the only bioactive mediator produced by this commensal organism. Functional depletion or selective neutralization studies will be necessary to determine the relative contribution of LP78 within the broader repertoire of *S. epidermidis*-derived factors.

Several limitations of this study should be considered when interpreting the results. First, the 4-week UV exposure protocol used here induces inflammatory and structural alterations in the skin but may not fully represent long-term chronic photodamage. Extended UV exposure models will therefore be required to more accurately recapitulate the complex processes underlying chronic photoaging. Second, the number of biological samples analyzed in both the *in vivo* and *in vitro* experiments was relatively limited, and larger sample sizes will be necessary to confirm the robustness and generalizability of these findings. Third, most mechanistic experiments were performed using neonatal human epidermal keratinocytes (NHEKs), a widely used model for studying keratinocyte innate immune responses. However, this system may not fully capture the biological properties of adult keratinocytes. Future studies incorporating adult keratinocytes and *ex vivo* human skin models will therefore be important to validate these observations in more physiologically relevant settings. Finally, although our data support a regulatory relationship between commensal-derived signals and TLR3-mediated inflammatory responses, additional work will be required to further delineate the molecular interactions and to determine their relevance in human photoaging.

In summary, our study suggests that *S. epidermidis* and its candidate bioactive component LP78 may function as endogenous regulators of UV-induced skin photoaging. By inducing TRAF1 and suppressing TLR3-mediated inflammatory signaling in keratinocytes, *S. epidermidis* limits epidermal senescence and reduces paracrine signals that promote dermal aging. These results highlight the potential importance of host-microbiota interactions in shaping inflammatory senescence in the skin and suggest that commensal-derived modulation of innate immune signaling may contribute to maintaining cutaneous homeostasis under chronic UV stress.

## Materials and methods

### Bacterial strains, cell lines, and reagents

*Staphylococcus epidermidis* was maintained in our laboratory. Neonatal human epidermal keratinocytes (NHEKs) and neonatal human fibroblasts were purchased from Lifeline Cell Technology. Immortalized human keratinocytes (HaCaT) were kindly provided by Dongqing Li (Key Laboratory of Basic and Translational Research on Immune-Mediated Skin Diseases, Chinese Academy of Medical Sciences). Primary murine keratinocytes (mKCs) and fibroblasts (mFBs) were isolated from the epidermis and dermis, respectively, of 2-3-day-old neonatal mice.

Lipopeptide 78 (LP78) was synthesized by Nanjing Gectide Biotechnology Co., LTD. Low-molecular weight fractions (≤10kDa) of *Staphylococcus epidermidis* culture supernatant (≤10kDa *S.epi*) were prepared as previously described (25). For topical application, ≤10kDa *S.epi* was incorporated into 2.5% (w/V) carboxymethylcellulose (CMC) hydrogel at a final concentration of 7ug/uL.

### UV-induced cell senescence

Cell were cultured to approximately 90% confluence, after which culture medium was removed and cells were washed three times with prewarmed phosphate-buffered saline (PBS). PBS was added to fully cover the cell monolayer. Cells designated for UV irradiation were exposed to UV light using TL20W/12 RS lamp (Philips). Non-irradiated control cells were shield with aluminum foil. For UVB irradiation, cells were exposed to a dose of 10mJ/cm^2^ (311nm), corresponding to an exposure time of approximately 5 seconds. For UVA irradiations, cells were exposed to 4 J/cm^2^ (365nm), corresponding to an exposure time of approximately 10 minutes. UV doses were measured using a UV energy detector to ensure accurate and consistent irradiation.

### Senescence-associated β-galactosidase staining and ROS detection

Senescence-associated β-galactosidase (SA-β-gal) activity was assessed using a Senescence β-galactosidase Staining kit (Cell Signaling Technology, #9860) according to the manufacturer’s instructions. Intracellular reactive oxygen species (ROS) levels were measured using the DCFH-DA probe (MedChemExpress, HY-D0940). Fluorescence was detected with excitation at 488 nm and emission at 525 nm.

### Long-term UV-induced photoaging mouse model

Wild-type (WT) and *Tlr3^−/−^* C57BL/6 mice were housed under specific pathogen-free (SPF) conditions at the East China Normal University (ECNU) Animal Facility. All animal procedures were approved by the ECNU Animal Care and Use Committee (Ethics Approval No. M20240311).

For induction of photoaging, the dorsal skin of 7-8-week-old mice was shaved and mice were randomly assigned to four groups: (1) no UV exposure with intradermal injection of tryptic soy broth (TSB), (2) no UV exposure with intradermal injection of ≤10kDa *S. epi*, (3) UV exposure with TSB injection, and (4) UV exposure with ≤10kDa *S. epi* injection. Mice received intradermal injections of 100 μL TSB or 100 μg of ≤10kDa *S. epi* (in 100μL) 2 hours prior to each UV exposure. Non-targeted areas were shielded with aluminum foil.

Mice were exposed to combined UVA and UVB irradiation three times per week for 4 consecutive weeks (See **Figure 1A**) using a custom-designed UV irradiation chamber with specific lamps providing UV light at 311nm (UVB) and 365nm (UVA). UV doses were monitored using a UV Energy meter. Cumulative UV doses were 16J/cm^2^ for UVA and 1.6J/cm^2^ for UVB after 100-minute exposure. Twenty-four hours after the final irradiation, mice were euthanized and dorsal skin samples were collected for histological, molecular and biochemical analyses. This model primarily reflects subacute UVB-induced skin damage, characterized by inflammatory responses and early tissue remodeling.

### siRNA transfection

Cells were seeded in 12-well plates and transfected at 50%-60% confluence with small interfering RNA (siRNA;10 nM) using jetPRIME transfection reagent (Polyplus, #101000046) according to the manufacturer’s instructions. Cells were harvested 24–48 hours post-transfection, depending on experimental requirements.

### Transwell co-culture assay

Transwell co-culture experiments were performed using young (passage ~P10) and senescent (passage ~P60) fibroblasts. Senescent fibroblasts were seeded in 10-cm dishes and grown to ~60% confluence, followed by stimulation with LP78 or ≤10kDa *S. epi* for 24 hours. Cells were then transferred to the upper chamber of Transwell inserts, while young fibroblasts were seeded in the lower chamber. Co-cultures were maintained for 48 hours with one medium change. Fibroblasts in the lower chamber were collected for downstream analyses.

### Preparation of conditioned medium containing SASP factors

Keratinocytes were seeded in 10-cm dishes and transfected at 50-60% confluence. Upon reaching ~90% confluence, cells were exposed to UVB (10mJ/cm^2^, approximately 5 s) or left untreated, followed by incubation in fresh medium for 48 h to generate conditioned medium containing SASP factors. Conditioned medium was collected, centrifuged at 2, 000 rpm for 20 minutes to remove cellular debris, and filtered through a 0.22μm membrane. Filtered medium was diluted with fresh Dulbecco’s Modified Eagle Medium (DMEM) to achieve a final serum concentration of 10%. For induction of fibroblast senescence, conditioned medium was applied to fibroblasts at ~60% confluence for 48 hours, with one medium change during incubation.

### Quantitative RT-PCR

Mouse skin tissues were homogenized using a tissue grinder (Shanghai Jingxin) in TRIzol reagent (Takara, 9109). Culture cells were lysed directly in TRIzol. Total RNA was extracted and reverse-transcribed using HiScript II Q RT SuperMix for qPCR (Novitzen, R223). Quantitative RT-PCR was performed using SYBR Green Master Mix (Yi Sheng, 11202) on a StepOnePlus real-time PCR system (Applied Biosystem). Relative gene expression was calculated using the 2^‐ΔΔCt^ method, with β-actin as the internal control. Primer sequences are provided in **Supplementary Table 1**.

### Enzyme-linked immunosorbent assay

To quantitatively assess SASP-associated cytokines in dorsal skin or cell cultures, cytokine levels were measured in tissue lysates using ELISA kits (R&D). As SASP factors are secreted proteins that may diffuse within the tissue microenvironment, ELISA provides a sensitive and quantitative approach for evaluating their overall abundance. Although immunofluorescence can reveal spatial localization of cytokine-producing cells, ELISA was used here to enable robust quantitative comparison of SASP cytokine levels between experimental groups.

### Western blotting

Total proteins were extracted using RIPA buffer and separated by SDS-PAGE (12-15% Tris-Glycine gels). Proteins were transferred to nitrocellulose membranes (PALL, 11327) and blocked with 5% nonfat milk for 1 hour at room temperature. Membranes were incubated overnight at 4 °C with primary antibodies against p16 (CST, 29271, 1:1000), p21 (CST, 37543, 1:1000), γ-H2Ax (Abcam, Ab11174, 1:2000), Traf1 (Santa, sc6253, 1:1000) and β-actin (Abclonal, AC004, 1:5000). After incubation with appropriate secondary antibodies, protein bands were visualized using an Odyssey two-color infrared imaging system. Band intensities were quantified using Image J software, and the protein levels were normalized to the corresponding loading control (β-actin).

### Histology and Masson’s trichrome staining

Formalin-fixed, paraffin-embedded tissue skin tissues were sectioned at ~5μm thickness. Hematoxylin and eosin (H&E) staining was performed as previously described (24). For Masson’s trichrome staining, deparaffinized sections were stained sequentially with hematoxylin, phosphomolybdic acid, ponceau acid fuchsin, and aniline blue according to standard protocols.

### Immunofluorescence staining

Cells were fixed in 4% paraformaldehyde for 15 minutes and permeabilized with PBS containing 0.2% Triton X-100 for 10 minutes. After blocking with 3% BSA for 30 minutes, cells were incubated with primary antibodies overnight at 4°C, followed by incubation with fluorescent secondary antibodies for 1 hour at room temperature. Nuclei were counterstained with DAPI. Images were captured using a Leica fluorescence microscope.

### Statistical analysis

Data are presented as mean ± SEM. All experiments were performed with three independent biological replicates per group unless otherwise indicated. Statistical analyses were performed using GraphPad Prism 8.0.1 software. Two-group comparisons were conducted using two-tailed Student’s *t*-tests. One-way ANOVA was applied for comparisons among multiple groups with a single factor, and Two-way ANOVA was used for experiments involving two or more variables. A *P* < 0.05 to be was considered statistically significant.

## Data Availability

The raw data supporting the conclusions of this article will be made available by the authors, without undue reservation.
