# Supplementary material for: Staphylococcus epidermidis prevents UV-induced skin aging by suppressing TLR3-mediated senescence
Source: Front Immunol. 2026 May 12;17:1796085. doi: 10.3389/fimmu.2026.1796085 (PMC13201166; doi:10.3389/fimmu.2026.1796085)
Supplement: Supplementary file 1 [file DataSheet1.docx]

**SUPPLEMENTARY MATERIAL**

**Supplementary Table**

Supplementary Table 1. Primers for the experiment

| Gene name | Primer sequence (5’ →3’) | Purpose |
| --- | --- | --- |
| Homo-*ACTIN* | F:CTTCGCGGGCGACGAT R:TAGGAATCCTTCTGACCCATGC | RT-PCR |
| Homo-*P53* | F:TGTGACTTGCACGTACTCCC R:ACCATCGCTATCTGAGCAGC | RT-PCR |
| Homo-*P21* | F:GACCTGTCACTGTCTTGTACCC R:TTGGAGTGGTAGAAATCTGTCATGC | RT-PCR |
| Homo-*P16* | F:GGAGGCCGATCCAGGTCAT R:CACCAGCGTGTCCAGGAAG | RT-PCR |
| Homo-*IL6* | F:GTTGTTGTTAATGGGCATTCC R:GTGTCCTAACGCTCATACTTT | RT-PCR |
| Homo-*IL1β* | F:ATGATGGCTTATTACAGTGGCAA R:GTCGGAGATTCGTAGCTGGA | RT-PCR |
| Homo-*TNFa* | F:CCTCTCTCTAATCAGCCCTCTG R:GAGGACCTGGGAGTAGATGAG | RT-PCR |
| Homo-*MMP1* | F:AAAATTACACGCCAGATTTGCC R:GGTGTGACATTACTCCAGAGTTG | RT-PCR |
| Homo-*P53* | F:CAGCACATGACGGAGGTTGT R:TCATCCAAATACTCCACACGC | PCR |
| Homo-*P21* | F:TGTCCGTCAGAACCCATGC R:AAAGTCGAAGTTCCATCGCTC | PCR |
| Homo-*P16* | F:ATGGAGCCTTCGGCTGACT R:GTAACTATTCGGTGCGTTGGG | PCR |
| Homo-*TLR3* | F:GGAGCCAGAATTGTGCCAGA R:AACACCCTGGAGAAAACTCTTT | PCR |
| Mus-A*ctin* | F:GGCTGTATTCCCCTCCATCG R:CCAGTTGGTAACAATGCCATGT | RT-PCR |
| Mus-*IL6* | F:CTGCAAGAGACTTCCATCCAGTT R:GGGAAGGCCGTGGTTGTC | RT-PCR |
| Mus-*IL1β* | F:TCCAGGATGAGGACATGAGCC R:GAACGTCACACACCAGCAGG | RT-PCR |
| Mus-*TNFa* | F:TCAAGGACTCAAATGGGCTTTC R:TGCAGAACTCAGGAATGGACAT | RT-PCR |
| Mus-*Tlr3* | F:GTGAGATACAACGTAGCTGACTG R:TCCTGCATCCAAGATAGCAAGT | RT-PCR |
| Mus-*siTlr3* | F: GCCUCUUUCUGAACAAUGUTT R: ACAUUGUUCAGAAAGAGGCTT | Gene knock-down |
| *Tlr3^–/–^* | AATTCATCAGTGCCATGAGTTT | *Tlr3^–/–^* Mouse genotyping |
| *WT* | GCAACCCTTTCAAAAACCAG | *Tlr3^–/–^* Mouse genotyping |
| *Tlr3^–/–^* mutant | GCCAGAGGCCACTTGTGTAG | *Tlr3^–/–^* Mouse genotyping |

**Supplementary Figures**


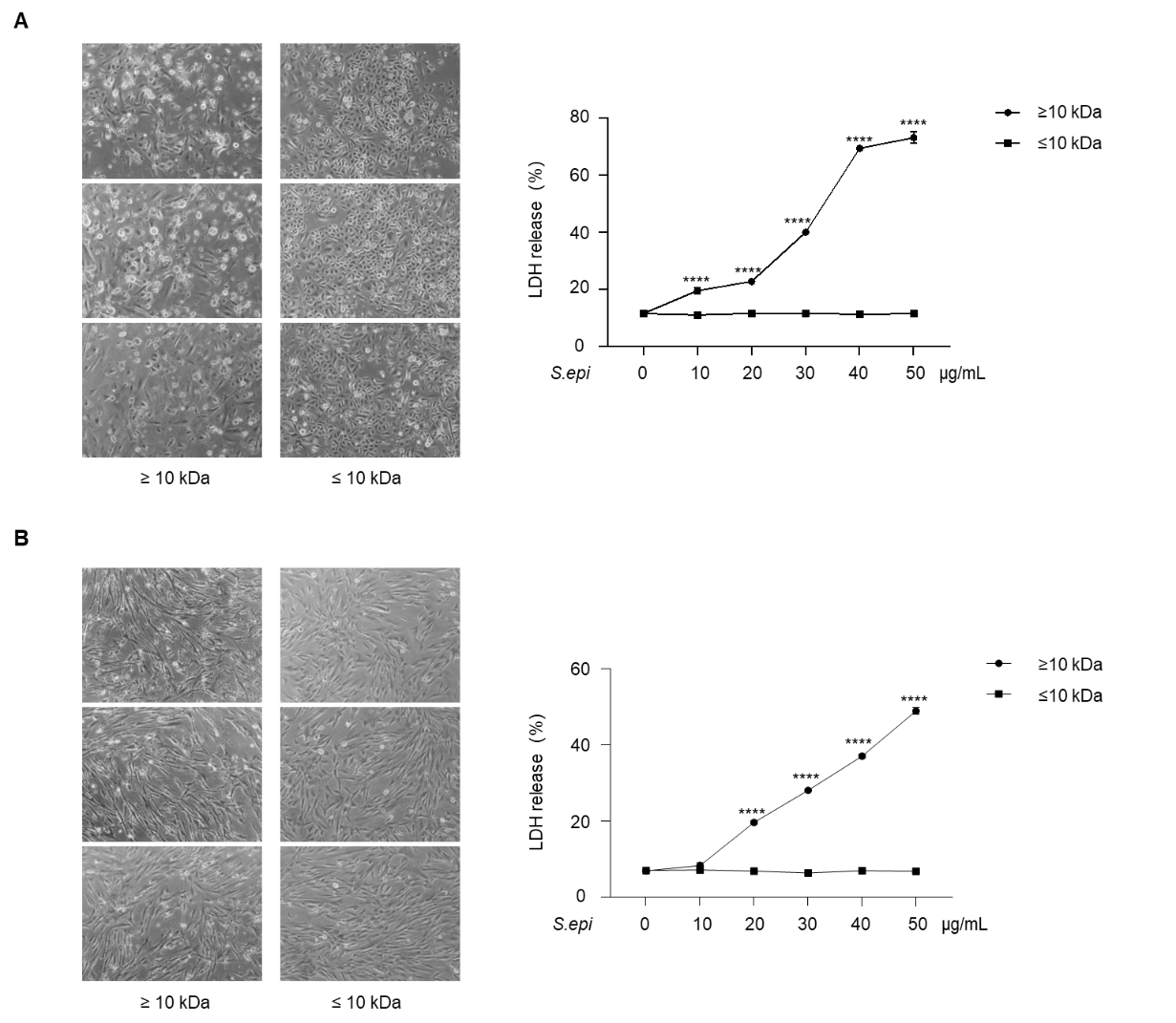


**Supplementary Figure 1. Cytotoxicity of *Staphylococcus epidermidis* conditioned medium to cultured keratinocytes and fibroblasts.** (A)When neonatal human epidermal keratinocytes (NHEKs) reached approximately 70% confluence, cells were treated with increasing concentrations of *Staphylococcus epidermidis*-conditional medium. Representative images were captured 24 hours after treatment, and cytotoxicity was assessed by measuring lactate dehydrogenase (LDH) release in the culture supernatant using an LDH assay kit. (B) Primary human fibroblasts at approximately 70% confluence were treated with different concentrations of *S. epidermidis* conditional medium under the same conditions. After 24 hours, Representative images were acquired, and LDH release in the supernatant was quantified. For each concentration, three replicates were included per experiment, and the experiment was independent repeated twice using separate cell cultures. Representative cell morphology images at 50 μg/mL *S.epi* conditional medium are shown. Data represent mean ± SEM with *n*=3. All the experiments have been repeated twice. Statistical significances were evaluated by Two-way ANOVA. *****p*<0.0001.


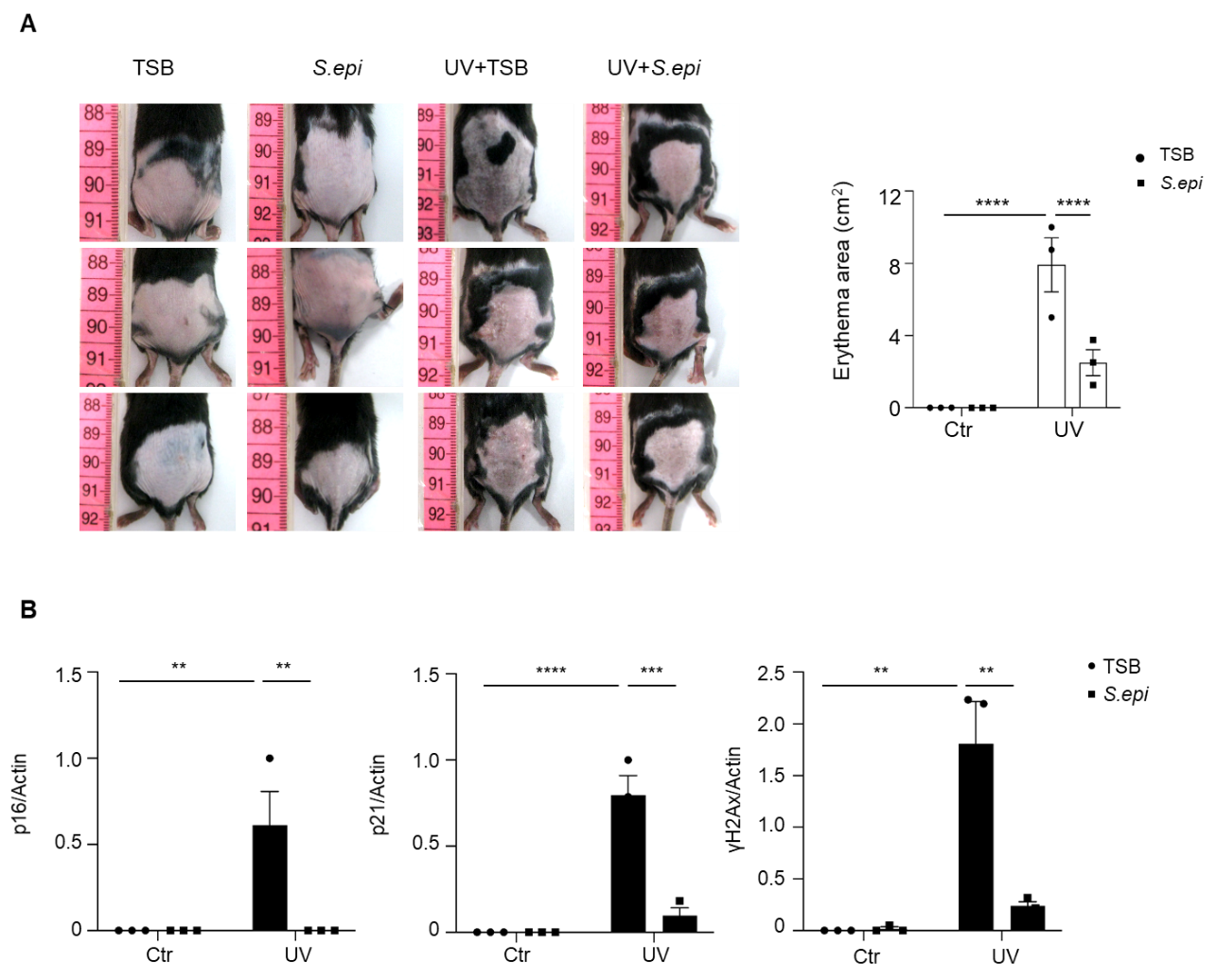


**Supplementary Figure 2. *Staphylococcus epidermidis* protect mice from UV-irradiation.** (A) Representative images of dorsal skin from mice received TSB or ≤10kDa *S. epi* injection showing the severity of UV-induced photoaging. Erythema area was quantified using ImageJ software. Differences in hair coverage among mice were not quantitatively assessed and may reflect variability in the hair cycle stage after shaving. The experiments have been repeated twice. (B) Densitometric analysis of protein bands shown in Figure 1E were performed using Image J software, and the quantified values were plotted using GraphPad Prism 8.0.1 software. Data represent mean ± SEM with *n*=3. Statistical significances were determined by Two-way ANOVA. ***p*<0.01, *****p*<0.0001.


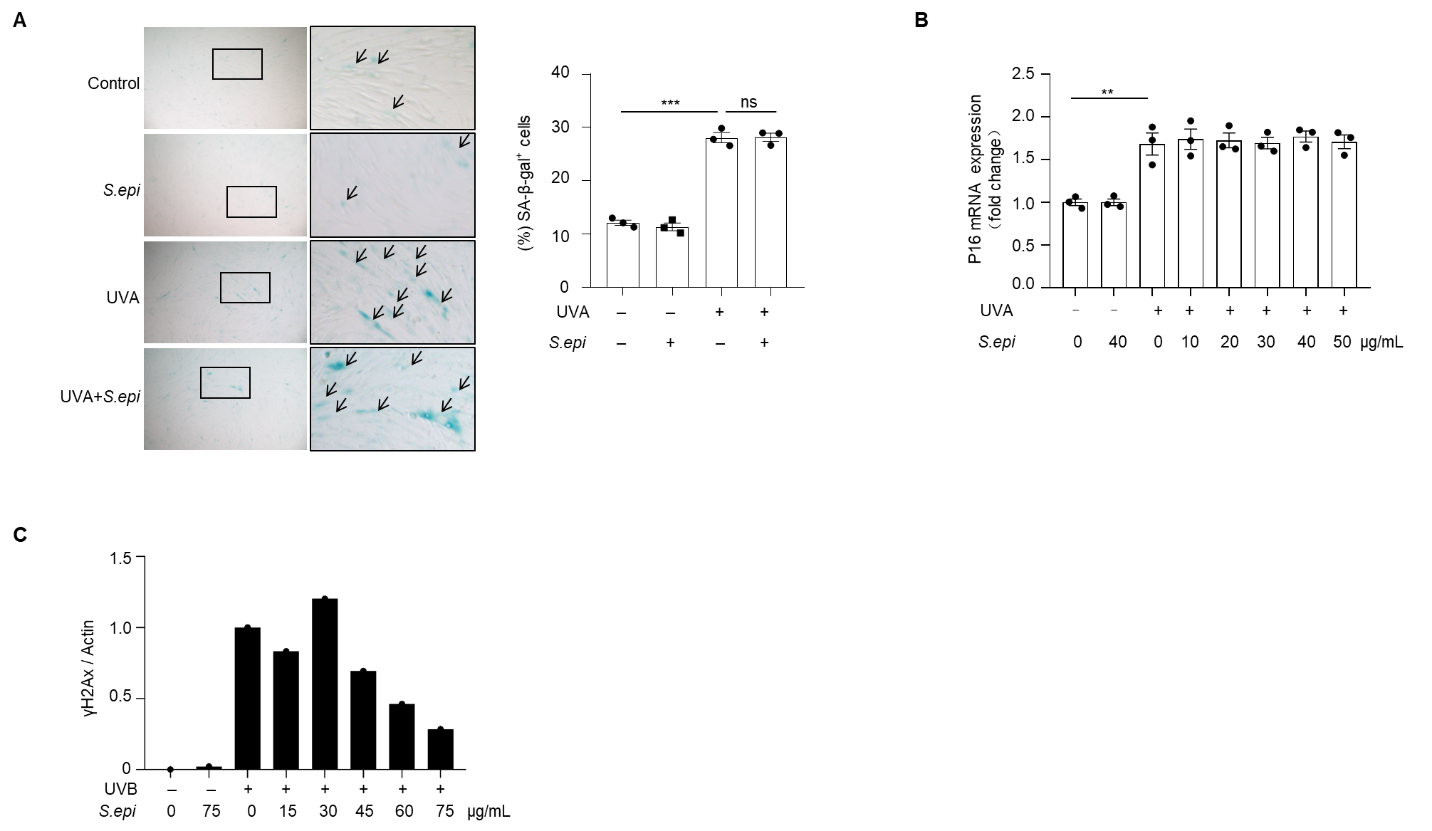


**Supplementary Figure 3. *Staphylococcus epidermidis* does not inhibit UVA-induced fibroblast senescence.** (A) Primary human fibroblasts at passage 8 were irradiated with 4J/cm^2^ UVA upon reaching 70% confluence, followed by treatment with 50μg/mL of the ≤10kDa *S. epi.* fraction. Senescence-associated β-galactosidase staining was performed 24 hours after irradiation, and the percentage of senescent cells was quantified by Image J. (B) Using the same experimental conditions as in (A), fibroblasts were treated with different concentrations of ≤10kDa *S. epi.* Following UVA irradiation. Total RNA was isolated 24 hours post-irradiation, and the expression of *P16* was detected by RT-PCR, with β-actin as the internal reference. (C) Densitometric analysis of protein bands shown in Figure 2D were performed using Image J software, and the quantified values were plotted using GraphPad Prism 8.0.1 software. Data represent mean ± SEM with *n*=3. All the experiments have been repeated three times. Statistical significances were evaluated by One-way ANOVA. ***p*<0.01, ****p*<0.001, n.s., no significance.


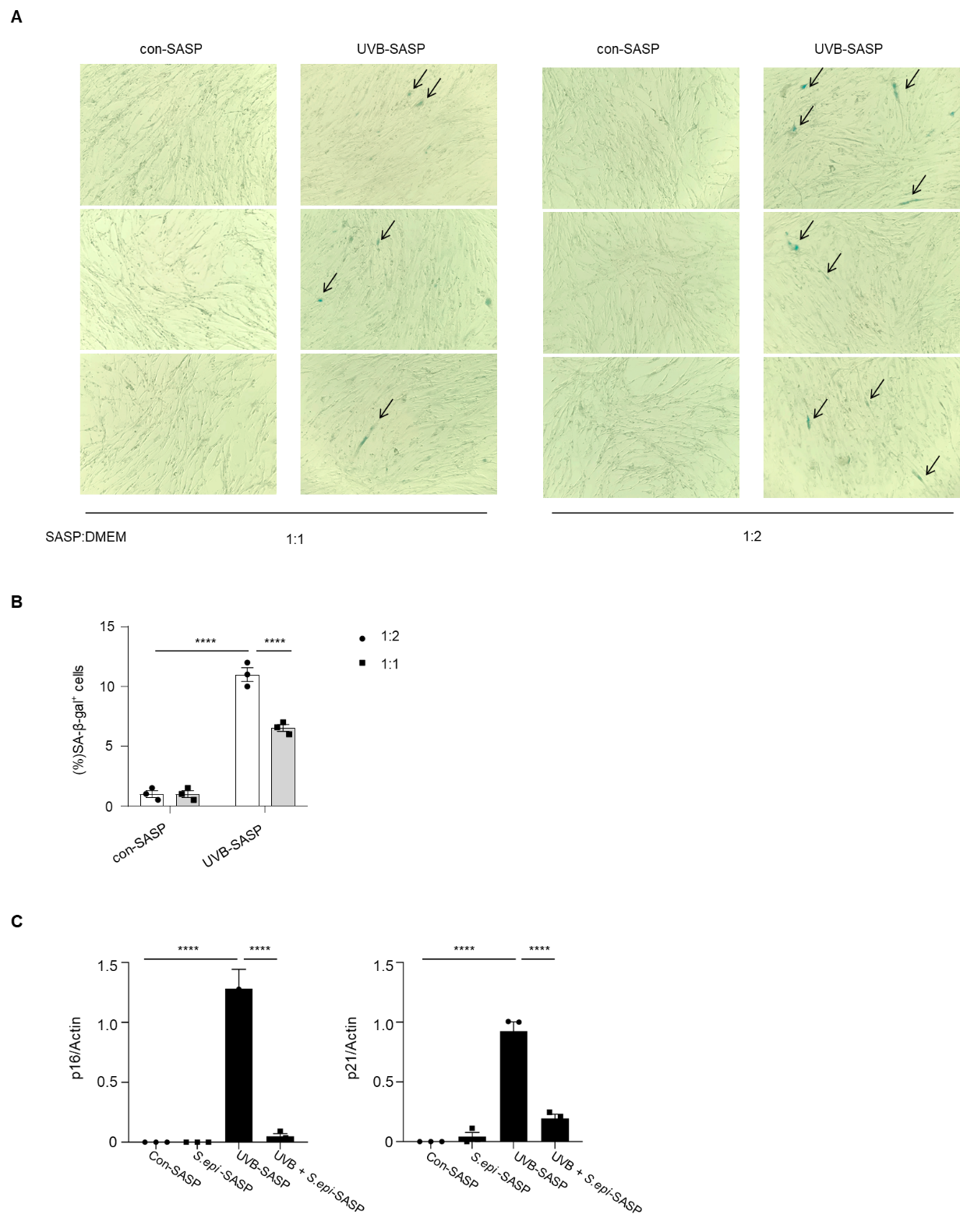


**Supplementary Figure 4. SASP factors secreted by UVB-exposed keratinocytes induce fibroblast senescence.** (A) Keratinocytes at approximately 70% confluence were irradiated with 10mJ/cm^2^ UVB and cultured for 48 hours. Conditioned medium containing senescence-associated secretory phenotype (SASP) factors was collected, centrifuged at 2,000 rpm for 20 minutes, and mixed with fresh DMEM medium at a 1:1 or 1:2 ration. The final serum concentration was adjusted to 10%, and the resulting media were used to culture primary human fibroblasts. After 48 hours, fibroblast senescence was assessed by β-galactosidase staining, with three independent replicates per group. (B) The percentage of senescent fibroblasts in (A) was quantified using ImageJ. (C) Densitometric analysis of protein bands shown in Figure 3C were performed using Image J software, and the quantified values were plotted using GraphPad Prism 8.0.1 software. Data represent mean ± SEM with *n*=3. The experiments have been repeated three times. Statistical significances were determined by Two-way ANOVA (B) or One-way ANOVA (C). *****p*<0.0001.


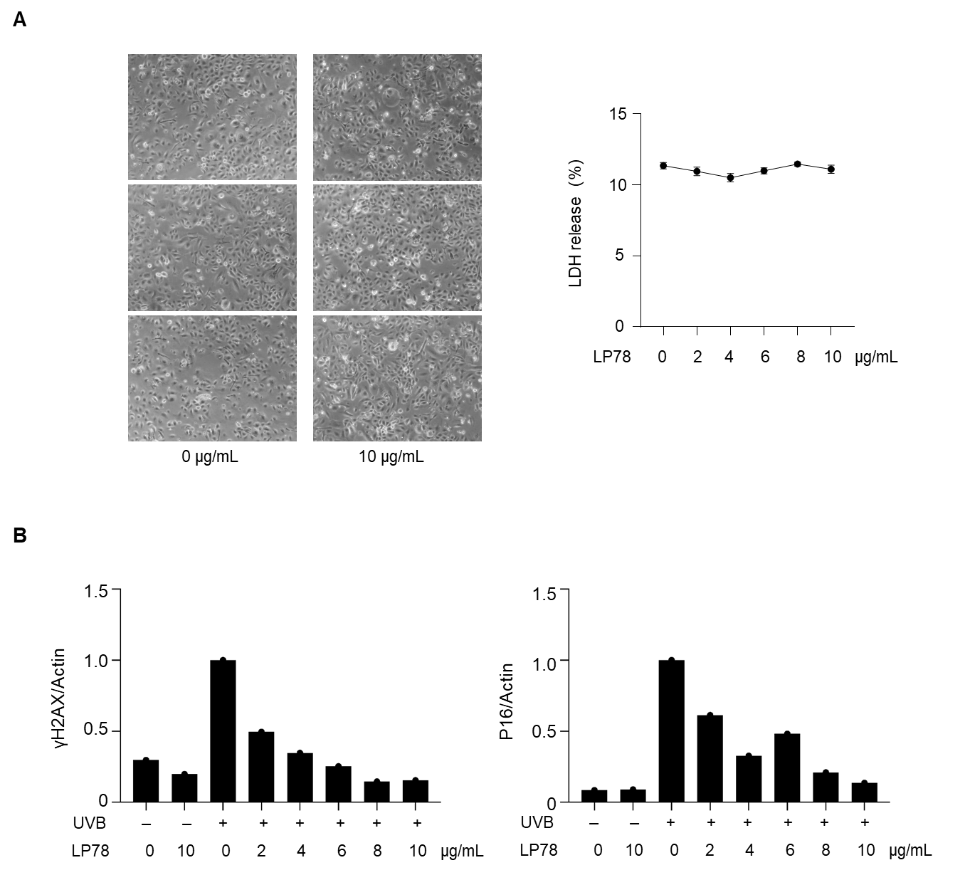


**Supplementary Figure 5. Cytotoxicity of LP78 in cultured keratinocytes.** (A) Keratinocyte at approximately 70% confluence were treated with different concentrations of LP78. Representative images were captured 24 hours after treatment, and cytotoxicity was assessed by measuring LDH release in the culture supernatant using an LDH assay kit. For each concentration, three independent replicates were performed, and representative cell morphology images at 10μg/mL LP78 are shown. (B) Densitometric analysis of protein bands shown in Figure 4B were performed using Image J software, and the quantified values were plotted using GraphPad Prism 8.0.1 software. Data represent mean ± SEM with *n*=3. The experiments have been repeated twice. Statistical significances were determined by One-way ANOVA.


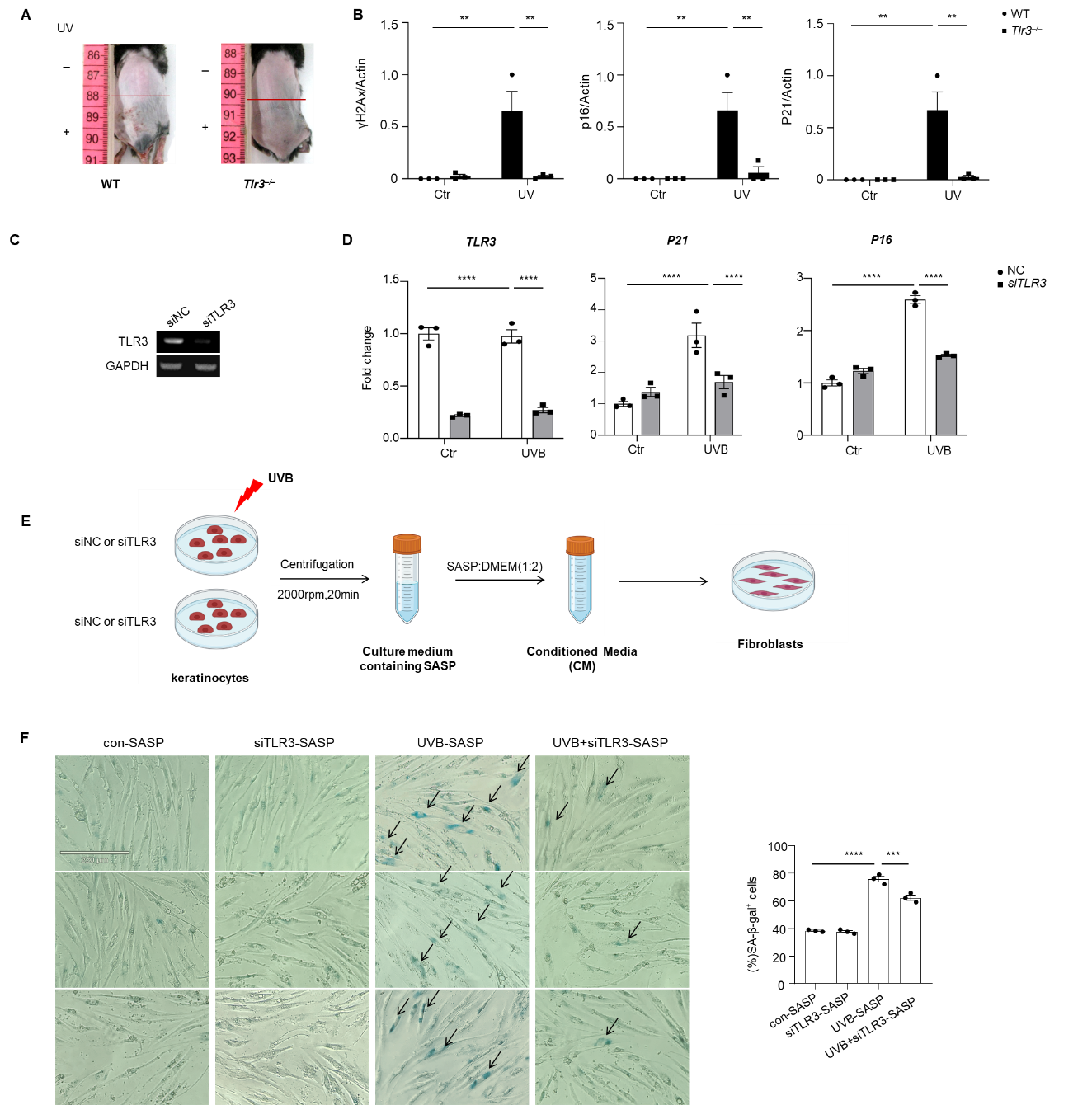


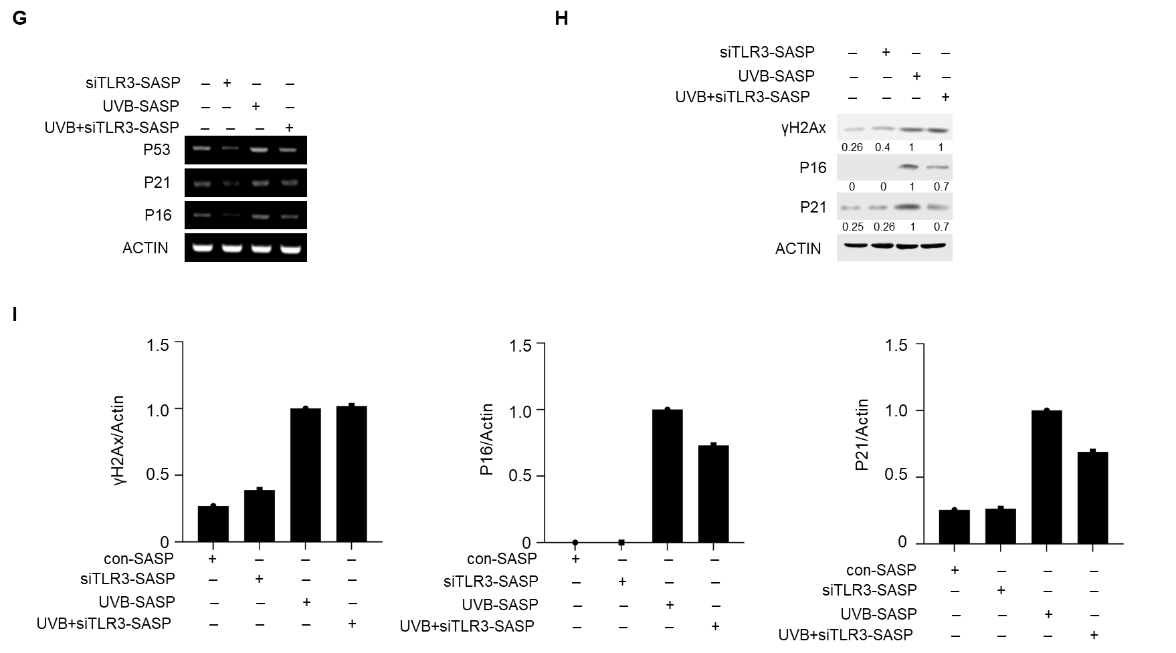


**Supplementary Figure 6. UVB-induced SASP factor production in keratinocytes is dependent on TLR3.** (A) Representative images of dorsal skin from WT and *Tlr3^‒/‒^* mice receiving intradermal injection of ≤10kDa *S. epi*, showing the severity of UV-induced photoaging. (B) Densitometric analysis of protein bands shown in Figure 5D were performed using Image J software, and the quantified values were plotted using GraphPad Prism 8.0.1 software. (C) Knockdown efficiency of *TLR3* was assessed by RT-PCR in keratinocytes transfected with control siRNA (siNC) or *TLR3*-targeting siRNA (si*TLR3*). (D) The expression of P21 and P16 in UVB-treated and untreated keratinocytes before and after TLR3 was silenced. (E) Schematic representation of the experimental design. Keratinocytes at approximately 60% confluence were transfected with si*TLR3* using jetPRIME. After 24 hours, cells were irradiated with 10mJ/cm^2^ UVB and cultured for an additional 48 hours. Conditioned medium containing SASP factors was collected and mixed with fresh DMEM medium at a 1:2 ratio with the final serum concentration adjusted to 10%. Primary human fibroblasts were then cultured with the mixed medium for 48 hours. (F) Fibroblasts cultured as described in (E) were subjected to senescence-associated β-galactosidase staining, and the percentage of senescent cells was quantified. (G) Fibroblasts cultured as described in (E) were analyzed for *P16*, *P21* and *P53* mRNA expression by RT-PCR, with β-actin used as the internal control. (H) Protein levels of P16, P21 and γH2Ax in fibroblasts cultured as described in (E) were determined by western blotting. (I) Densitometric analysis of protein bands shown in (H) were performed using Image J software, and the quantified values were plotted using GraphPad Prism 8.0.1 software. Data represent mean ± SEM with *n*=3. The experiments have been repeated twice or three times. Statistical significances were determined by Two-way ANOVA (B & D) or One-way ANOVA (F & I). ***p*<0.01, ****p*<0.001, *****p*<0.0001.


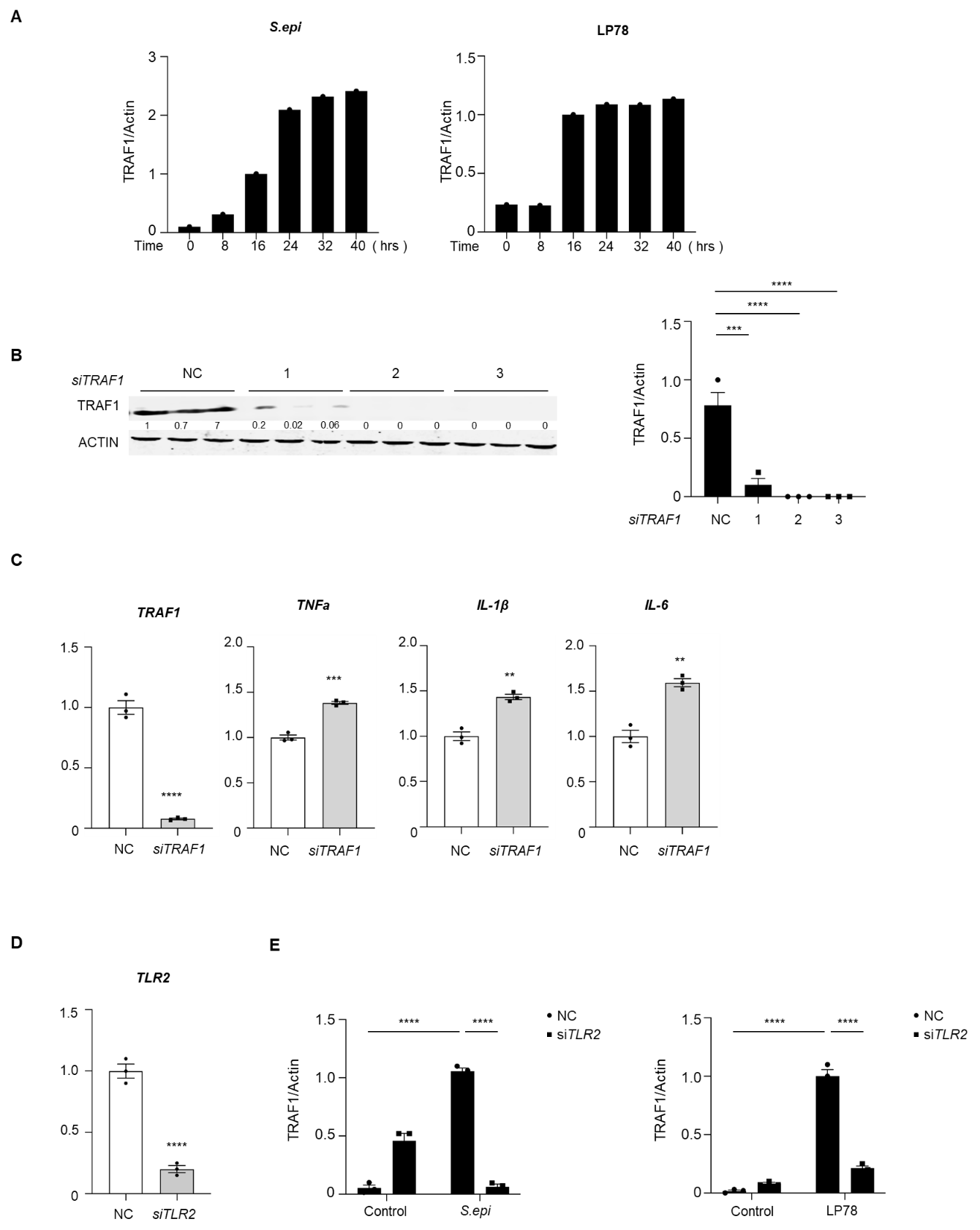


**Supplementary Figure 7. The expression of *TRAF1* and *TLR2* in NEHKs.** (A) Densitometric analysis of protein bands shown in Figure 6B were performed using Image J software, and the quantified values were plotted using GraphPad Prism 8.0.1 software. (B) Neonatal human epidermal keratinocytes (NHEKs) were transfected with *TRAF1*-targeting siRNA (*siTRAF1*) using jetPRIME at approximately 60% confluence. After 24 hours, cell lysates were collected and TRAF1 protein levels were analyzed by western blotting. Densitometric analysis of protein bands were performed using Image J software, and the quantified values were plotted using GraphPad Prism 8.0.1 software. (C) NHEKs were transfected with *siTRAF1* as described in (B). After 24 hours, total RNA was isolated, and the mRNA the expression of *TRAF1*, *TNFα*, *IL-1β* and *IL-6* was quantified by RT-PCR. (D) NHEKs were transfected with *TLR2*-targeting siRNA (*siTLR2*) using jetPRIME at approximately 60% confluence. After 24 hours, total RNA was isolated, and *TLR2* mRNA expression was assessed by RT-PCR. (E) Densitometric analysis of protein bands shown in Figure 7A were performed using Image J software, and the quantified values were plotted using GraphPad Prism 8.0.1 software. Data represent mean ± SEM with *n*=3. The experiments have been repeated three times. Statistical significances were determined by One-way ANOVA (A & B), two-tailed *t* tests (C & D) or Two-way ANOVA (E). ***p*<0.01, ****p*<0.001, *****p*<0.0001.
